# Antibiotic-associated adverse events in bone and joint infections: a FAERS pharmacovigilance study

**DOI:** 10.3389/fmed.2026.1887555

**Published:** 2026-07-24

**Authors:** Haoping Dai, Hongtao Li, Changming Xiao

**Affiliations:** Department of Spine Surgery, The Affiliated Traditional Chinese Medicine Hospital, Southwest Medical University, Luzhou, Sichuan, China

**Keywords:** adverse drug events, antibiotics, bone and joint infection, FAERS, pharmacovigilance

## Abstract

**Background:**

Bone and joint infections (BJI) require prolonged antibiotic therapy that may amplify cumulative toxicity risk, yet indication-contextualized safety data remain sparse.

**Methods:**

Using the FDA Adverse Event Reporting System (FAERS, 2004Q1-2025Q4; 20,006,981 deduplicated cases), we identified 9,072 cases involving 17 BJI-target antibiotics. Four disproportionality measures, namely reporting odds ratio (ROR), proportional reporting ratio (PRR), information component (IC), and empirical Bayes geometric mean (EBGM), were applied against the full FAERS database. We then performed BJI within-cohort contextualization, primary-suspect-only (PS-only) sensitivity analysis, exploratory XGBoost machine learning, and Weibull time-to-onset (TTO) modeling, in accordance with the READUS-PV reporting framework.

**Results:**

Of 153 drug-adverse drug event (ADE) pairs, 103 met the prespecified four-method robust full-FAERS signal criterion; 28 were retained after BJI within-cohort contextualization. Indication-supported signals included vancomycin-nephrotoxicity (within-cohort ROR 3.30, 95% CI 2.95–3.70), linezolid-hematologic toxicity (3.65, 3.21–4.16), ceftaroline-hematologic toxicity (4.81, 3.42–6.77), and ertapenem-neurotoxicity (4.76, 3.50–6.47). Several strong full-FAERS signals (e.g., fluoroquinolone-tendon and broad-spectrum-C. difficile infection) were attenuated within the BJI cohort or under PS-only restriction, highlighting the role of prescribing pattern and reporting-role confounding. Weibull modeling indicated predominantly early-onset reporting patterns.

**Conclusion:**

Multi-method, indication-contextualized FAERS analysis distinguishes BJI-supported from general reporting signals and provides a transparent framework for prioritizing monitoring during prolonged antibiotic therapy. Findings are hypothesis-generating and require validation in controlled observational studies. They prioritize safety signals rather than providing estimates of incidence, absolute risk, causality, dose-response, or renal-function effects.

## Introduction

Bone and joint infections (BJI), encompassing osteomyelitis, prosthetic joint infections, and device-related infections, are severe complications requiring prolonged systemic antibiotic therapy typically lasting 6–12 weeks (1–3). The extended treatment duration substantially increases the risk of cumulative exposure-related toxicity compared with short-course antimicrobial regimens, as antibiotic-associated adverse events have been shown to increase with longer treatment duration (4).

The adverse event profiles of individual antibiotics are well characterized in general populations and include vancomycin-associated nephrotoxicity, linezolid-induced thrombocytopenia, and fluoroquinolone-related tendon disorders as established safety concerns (5–7). However, systematic evaluation of antibiotic safety specifically in BJI patients using large-scale post-market surveillance databases remains limited.

The FDA Adverse Event Reporting System (FAERS) is one of the largest spontaneous reporting databases for post-market drug safety surveillance (8). Disproportionality analysis methods (including the reporting odds ratio, proportional reporting ratio, information component, and empirical Bayes geometric mean (9–12)) enable systematic signal detection (13, 14). Recent applications of machine learning in pharmacovigilance, particularly gradient boosting with SHAP interpretation, have complemented traditional approaches (15–17).

Few studies have systematically evaluated antibiotic safety signals in an indication-enriched BJI cohort while distinguishing general FAERS reporting enrichment from signals that persist within a BJI-specific background. This distinction is clinically important because many antibiotic-ADE associations are already well recognized in general populations, whereas their relative prominence during prolonged BJI treatment may be shaped by indication, treatment duration, comorbidity, surgical complexity, and concomitant therapy. To our knowledge, this is the first FAERS pharmacovigilance study to (i) prespecify a multi-method robust signal definition for BJI-target antibiotics across 22 years, (ii) directly contrast full-FAERS signals against an indication-enriched BJI background to separate prescribing-related reporting enrichment from BJI-supported signals, and (iii) integrate primary-suspect-only sensitivity testing with Weibull time-to-onset (TTO) characterization in a single READUS-PV-compliant framework. Accordingly, the study aimed to identify antibiotic-ADE reporting signals using four complementary disproportionality methods against the full FAERS database, contextualize these signals within the BJI cohort, and characterize supportive patterns using PS-only sensitivity analysis, exploratory machine learning, and Weibull TTO modeling.

## Methods

This study was reported in accordance with the Reporting of a Disproportionality Analysis for Drug Safety Signal Detection Using Individual Case Safety Reports in PharmacoVigilance (READUS-PV) recommendations where applicable (18).

### Data source

This retrospective pharmacovigilance study utilized publicly available quarterly ASCII data extracts from the FDA Adverse Event Reporting System (FAERS), spanning 2004Q1 through 2025Q4 (88 quarterly files; corrupted downloads were re-obtained and the final dataset covered all 88 quarters). All data were de-identified; institutional review board approval was not required (19).

### Cohort selection and preprocessing

Case identification followed a three-step pipeline. The 17 target antibiotics were selected on the basis of IDSA prosthetic joint infection management guidelines (2) and IDSA osteomyelitis consensus recommendations, recent BJI antimicrobial stewardship literature, and inclusion of newer agents (cefiderocol, dalbavancin, tedizolid) approved during the study period to capture emerging post-market safety signals. First, the DRUG table was searched for these 17 target antibiotics commonly used in BJI management (vancomycin, daptomycin, linezolid, tedizolid, rifampicin, ciprofloxacin, levofloxacin, moxifloxacin, trimethoprim-sulfamethoxazole, ceftriaxone, cefazolin, ceftaroline, piperacillin-tazobactam, meropenem, ertapenem, dalbavancin, and cefiderocol; Supplementary Table S1) using standardized active ingredient matching (prod_ai field) and case-insensitive regular expressions on the drugname field. Only drug records coded as primary suspect (PS) or secondary suspect (SS) were retained; concomitant (C) and interacting (I) roles were excluded. Restriction to PS and SS roles follows the conservative approach recommended by READUS-PV (18) for primary signal detection in indication-specific analyses; sensitivity analyses including C/I roles are reserved for future targeted re-analyses where indication vs. suspect attribution can be case-by-case adjudicated.

Second, the INDI (indication) table was used to identify cases with BJI-related indications through a two-tier strategy. Tier 1 (high specificity) included cases where a target antibiotic had an indication matching established BJI MedDRA preferred terms, including osteomyelitis, device related infection, intervertebral discitis, post procedural infection, septic arthritis, implant site infection, medical device site joint infection, device related sepsis, joint infection, bone infection, arthritis infective, osteomyelitis bacterial, and osteomyelitis chronic (complete frequency list in Supplementary Table S2). Tier 2 (contextual) included cases where the target antibiotic had a generic infection indication (e.g., infection, bacterial infection, staphylococcal infection) and another drug in the same case had an orthopedic procedure indication (e.g., arthroplasty, fracture, spinal fusion). Several additional Tier 1 terms (prosthetic joint infection, staphylococcal bone infection, orthopedic device infection) returned zero matches, likely reflecting MedDRA coding variability. Tier 1 terms accounted for 8,944 cases (98.6% of the cohort), while Tier 2 contributed 128 additional cases (1.4%), indicating that the cohort is overwhelmingly defined by direct BJI indication terms. A stricter drug-linked Tier 1 analysis was also applied, in which the target-antibiotic drug record had to be explicitly linked to a Tier 1 BJI indication through the FAERS indi_drug_seq field. The inclusive *N* = 9,072 cohort is presented as the main cohort, retained to preserve sample size for the exploratory machine-learning model and the within-cohort contextualization, with the drug-linked Tier 1 cohort (*N* = 8,309, which additionally excluded 763 cases that could not be explicitly drug-linked—a larger and more conservative exclusion than removing the 128 contextual Tier 2 cases alone) presented as a pre-specified confirmatory analysis; the two cohorts yielded concordant conclusions, with all four headline signals essentially unchanged (absolute ROR change ≤ 10.7%; Supplementary Table S3).

Third, report deduplication followed FDA-recommended case-versioning rules. (1) All reports sharing the same CASEID were grouped; (2) the record with the highest primaryid was retained; (3) ties were resolved by the most recent FDA_DT; (4) remaining ties were resolved by fewest missing fields. After deduplication, the final cohort comprised 9,072 unique cases with 17,092 drug-case records (PS 7,396; SS 9,696).

All analyses were performed in Python 3.12.8 using pandas 2.3.3, NumPy 2.4.4, SciPy 1.17.1, XGBoost 3.2.0, SHAP 0.51.0, scikit-learn 1.8.0, and matplotlib 3.10.8.

### Adverse event classification

Adverse events from the REAC table were classified into nine clinically relevant ADE categories based on Medical Dictionary for Regulatory Activities (MedDRA, version 27.0) preferred terms (20), namely nephrotoxicity, hepatotoxicity, hematologic toxicity, Clostridioides difficile infection (CDI), neurotoxicity, tendon/musculoskeletal disorders, cardiovascular toxicity, skin/allergic reactions, and ototoxicity (complete PT mapping in Supplementary Table S4). For the operational within-cohort analyses, tendon/musculoskeletal disorders were restricted to tendon rupture/tendinitis-type preferred terms, whereas rhabdomyolysis, myopathy, blood creatine phosphokinase increased, myositis, myalgia, muscular weakness, and related muscle-injury terms were handled separately as a daptomycin-associated myotoxicity construct; consequently, the within-cohort Tendon/MSK exposed-event counts in Supplementary Table S5 exclude these myotoxicity preferred terms. The unrestricted preferred-term breakdown for daptomycin (the principal agent for which this operational restriction matters) is provided in Supplementary Table S6. FAERS reaction terms also include medication errors, product use issues, and lack of efficacy reports; these were excluded from the nine ADE categories but are present in the overall reaction frequency tables, as reflected in FAERS reporting structures (19). Drug fever, drug-induced hypersensitivity syndrome (beyond the SJS/TEN/DRESS terms already represented within the Skin/Allergy category), and gastrointestinal disturbances other than CDI were not separately analyzed because of (i) preferred-term coding heterogeneity that made prespecified pool construction unreliable in pilot exploration and (ii) low BJI-relevant cumulative incidence; QT prolongation and other arrhythmic events are captured within the cardiovascular category. The corresponding within-BJI myotoxicity disproportionality and active-comparator results for daptomycin are reported in Supplementary Tables S7 and S8.

### Disproportionality analysis

For each of 153 drug-ADE category pairs (17 antibiotics × 9 categories), 2 × 2 contingency tables were constructed using the entire deduplicated FAERS database (N = 20,006,981) as the reference population. Cell counts therefore reflect drug-ADE co-occurrence across all FAERS reports, not limited to the 9,072 BJI cohort cases. A Haldane-Anscombe continuity correction of 0.5 was added to all four cells of 2 × 2 tables whenever any cell count was zero; otherwise raw counts were used. Four measures were calculated, namely ROR with 95% CI, PRR with 95% CI, IC with 95% credible interval, and EBGM with EB05 lower bound (9–12). Signal thresholds were as follows. ROR lower CI > 1 and a ≥ 3; PRR ≥2, **χ**^**2**^ **≥4**, and a ≥3; IC lower CI > 0; and EB05 ≥2. The PRR criterion of χ^2^ ≥4 follows the Evans signal-detection rule (10) and represents the conventional integer rounding of the exact chi-square critical value at the two-sided 5% significance level for one degree of freedom (χ^2^ = 3.84 at α = 0.05, df = 1). A drug-ADE pair was considered a robust full-FAERS signal when all four methods simultaneously met their predefined thresholds. Benjamini-Hochberg (BH) false discovery rate correction was applied to the 153 full-FAERS pairs at the 5% level (21); within-cohort, PS-only, and bacterial-pneumonia comparator analyses were contextual sensitivity analyses applied to an already-defined candidate set of signals and were therefore not separately FDR-adjusted (consistent with the hierarchical reporting framework recommended by READUS-PV18).

### Within-cohort contextualization

To contextualize full-FAERS signals within the BJI cohort, a secondary within-cohort disproportionality analysis was performed using the 9,072 BJI cases as the reference population. A full-FAERS robust signal was considered retained in the BJI context when the within-cohort ROR lower 95% CI exceeded 1.0 and the exposed-event count was at least 3. This asymmetric standard relative to the full-FAERS four-method criterion was prespecified because the within-cohort analysis was intended as contextualization in a smaller indication-enriched cohort rather than independent large-scale signal discovery. Within-cohort PRR, IC, and EBGM estimates are provided in Supplementary Table S5 columns; applying a within-cohort four-method robust criterion (mirroring the full-FAERS definition) would have retained approximately 22 of the 28 BJI-retained signals, with the difference predominantly attributable to small exposed-event counts (a < 10) limiting PRR/IC/EBGM statistical power in the smaller indication-enriched cohort. These findings are described as BJI-retained or BJI-supported reporting signals, distinguishing core signals supported by strict Tier 1, PS-only, and active-comparator sensitivity analyses from sparse exploratory signals.

To externally validate the indication-specificity of the within-cohort signals, a comparator analysis was performed using a bacterial-pneumonia (BP) cohort (*n* = 3,581 unique cases) defined via 26 BP-related MedDRA preferred terms (Pneumonia, Pneumonia bacterial, Lower respiratory tract infection, and related variants; viral, fungal, Pneumocystis, COVID-19, hypersensitivity, organizing, idiopathic interstitial, radiation, eosinophilic, and community-acquired pneumonia were excluded; see Table 1 footnote for the cohort construction details). Within-BP-cohort 2 × 2 contingency tables for the four prioritized BJI-supported reporting signals were constructed using the same continuity-corrected ROR estimator and the identical ADE PT pools (Supplementary Table S4). The BP cohort was selected as the comparator because it represents an alternative indication with substantial antibiotic exposure but markedly shorter typical treatment duration (approximately 5–14 days vs. 6–12 weeks for BJI), permitting empirical separation of duration-dependent from acute-dose-related toxicities (22). As a long-course comparator, an infective-endocarditis (IE) cohort (*n* = 1,500) was additionally constructed from 26 endocarditis-spectrum MedDRA preferred terms (non-infective variants excluded) using the identical drug-role restriction, ADE preferred-term pools, and continuity-corrected within-cohort ROR procedure as the BP comparator (Supplementary Table S9).

**Table 1:** External validation of within-cohort signals against a bacterial-pneumonia (BP) comparator cohort.

Drug—ADE category	BJI ROR (95% CI)	IE ROR (95% CI)	BP ROR (95% CI)	BJI/IE ratio	BJI/BP ratio	Interpretation
Linezolid**—**hematologic	3.65 (3.21–4.16)	2.75 (2.00–3.78)	1.94 (1.55–2.43)	1.33	1.88	Duration-dependent (elevated in long-course BJI and IE; lower in short-course BP)
Vancomycin**—**nephrotoxicity	3.30 (2.95–3.70)	2.71 (1.84–3.98)	4.83 (3.74–6.24)	1.22	0.68	Class/acute-dose effect (comparable across BJI, IE and BP)
Ceftaroline**—**hematologic	4.81 (3.42–6.77)	2.66 (1.81–3.92)	0.86 (0.41–1.79)	1.81	5.59	Duration-dependent (elevated in BJI and IE; absent in short-course BP)
Ertapenem**—**neurotoxicity	4.76 (3.50–6.47)	1.56 (0.21–11.78)†	1.11 (0.58–2.14)	—	4.26	BJI signal; IE sparse (a = 1) and not interpretable; short-course BP low

The BJI/BP ratio quantifies the relative magnitude of indication-contextualized signal enrichment in BJI vs. an alternative indication with substantially shorter typical treatment duration. A long-course infective-endocarditis (IE) comparator is also reported (Supplementary Table S9); the per-drug exposed-case counts previously shown as columns are provided in Supplementary Table S1 and the comparator tables.

### Exploratory machine-learning association analysis

An XGBoost gradient boosting classifier was developed as an exploratory association analysis to identify features associated with reported severe outcomes in FAERS. The outcome was defined as death or life-threatening events (case-level union of death (DE) and life-threatening (LT) outcome codes; *n* = 1,401, 15.4% of cohort). Hospitalization was excluded from the outcome definition to focus on the most clinically severe events and to avoid the high baseline hospitalization rate (50.8%) that would reduce discriminative power. The feature set comprised 38 variables spanning antibiotic exposure indicators, patient demographics, treatment complexity, and reporter characteristics; the complete feature list, coding scheme, and missing-data handling are provided in Supplementary Tables S10, S11. The 38 features were prespecified into three categories (biologic (age, sex, body weight; *n* = 5), drug exposure (17 antibiotic indicators, intravenous and oral routes, total drugs, therapy duration, indication context; *n* = 25), and reporting metadata [report year, US report indicator, reporter type, total reactions, has-PS-role indicator; *n* = 8)] to enable interpretation of feature-importance rankings in causal vs. reporting-behavior terms. Hyperparameters and validation settings are summarized in Supplementary Table S12. Model performance was estimated using stratified five-fold cross-validation; class imbalance was addressed using scale_pos_weight set to the negative-to-positive class ratio (5.48). SHAP values were used for *post hoc* feature interpretation. The model was not calibrated for individual-level prediction and was used only to describe associations within the reporting dataset.

### Time-to-onset analysis

Time-to-onset (TTO) was calculated as the interval between the adverse event date (DEMO.event_dt) and therapy start date (THER.start_dt), retaining records with 0 < TTO ≤ 3,650 days. For cases with multiple target antibiotics, each drug-therapy episode generated a separate TTO record via the primaryid linkage; consequently, per-drug TTO record counts may sum to more than the overall unique record count. TTO analyses were restricted to records with complete and valid date information, and results were interpreted descriptively because timing completeness in spontaneous reporting databases is incomplete and potentially selective. A baseline comparison of TTO-evaluable cases (those with both valid event_dt and valid start_dt; *n* = 4,761) vs. the missing-date subset (*n* = 4,311) is provided in Supplementary Table S13 to characterize selective-availability bias; the published TTO analytic subset (*n* = 4,168) reflects further filtering to 0 < TTO ≤ 3,650 days. TTO-evaluable cases were modestly older (median 67 vs. 64 years), more likely to have a serious outcome (64.6% vs. 51.6%), and more frequently physician-reported, consistent with documentation-completeness selection rather than systematic clinical divergence. The Weibull distribution was fitted using maximum likelihood estimation with location fixed at zero (23). The shape parameter (α) was estimated with 95% CI via 500 bootstrap resamples. The 500-resample count follows Sauzet et al. (23); *post-hoc* sensitivity re-estimation with 2,000 resamples narrowed CI widths by less than 5% across all 17 evaluable antibiotics and did not change failure-type classification (early failure, indeterminate, or wear-out) for any antibiotic, supporting the adequacy of the prespecified baseline. Classification was applied as follows. Early failure when α upper CI < 1; wear-out only when α lower CI >1; indeterminate when CI crossed 1.0. Sensitivity analyses retaining only the earliest valid onset per case and the earliest valid onset per drug-case were performed to evaluate whether repeated drug-therapy episodes drove the Weibull interpretation (Supplementary Table S14).

### Sensitivity analysis

A primary-suspect-only (PS-only) sensitivity analysis was performed to assess whether key disproportionality signals were driven by primary suspect attribution or by secondary suspect / concomitant drug reporting. The drug-record set was restricted to PS role only, yielding 7,389 BJI cases that retained at least one PS record for a target antibiotic (an 18.6% reduction from the full 9,072-case cohort). The top 20 drug-ADE signals (ranked by full-FAERS ROR) were selected for PS-only re-evaluation; this scope (rather than all 103 robust signals) was prespecified to focus on the highest-magnitude signals where PS-only attenuation would carry the most interpretive weight. Within-cohort 2 × 2 contingency tables were reconstructed using the PS-only BJI subset (*n* = 7,389) as the reference population, with a continuity correction of 0.5 applied. Pairs with PS-only exposed-event count a = 0 were retained but flagged as unstable (Supplementary Table S15). Drug-role attribution for prioritized signals was summarized, and exploratory active-comparator analyses were conducted within the BJI cohort using mutually exclusive exposed cases, including vancomycin versus daptomycin, daptomycin versus vancomycin, linezolid versus vancomycin, ceftaroline versus ceftriaxone, and ertapenem versus meropenem comparisons (Supplementary Tables S7, S8).

## Results

### Cohort characteristics

**Figure 1:**
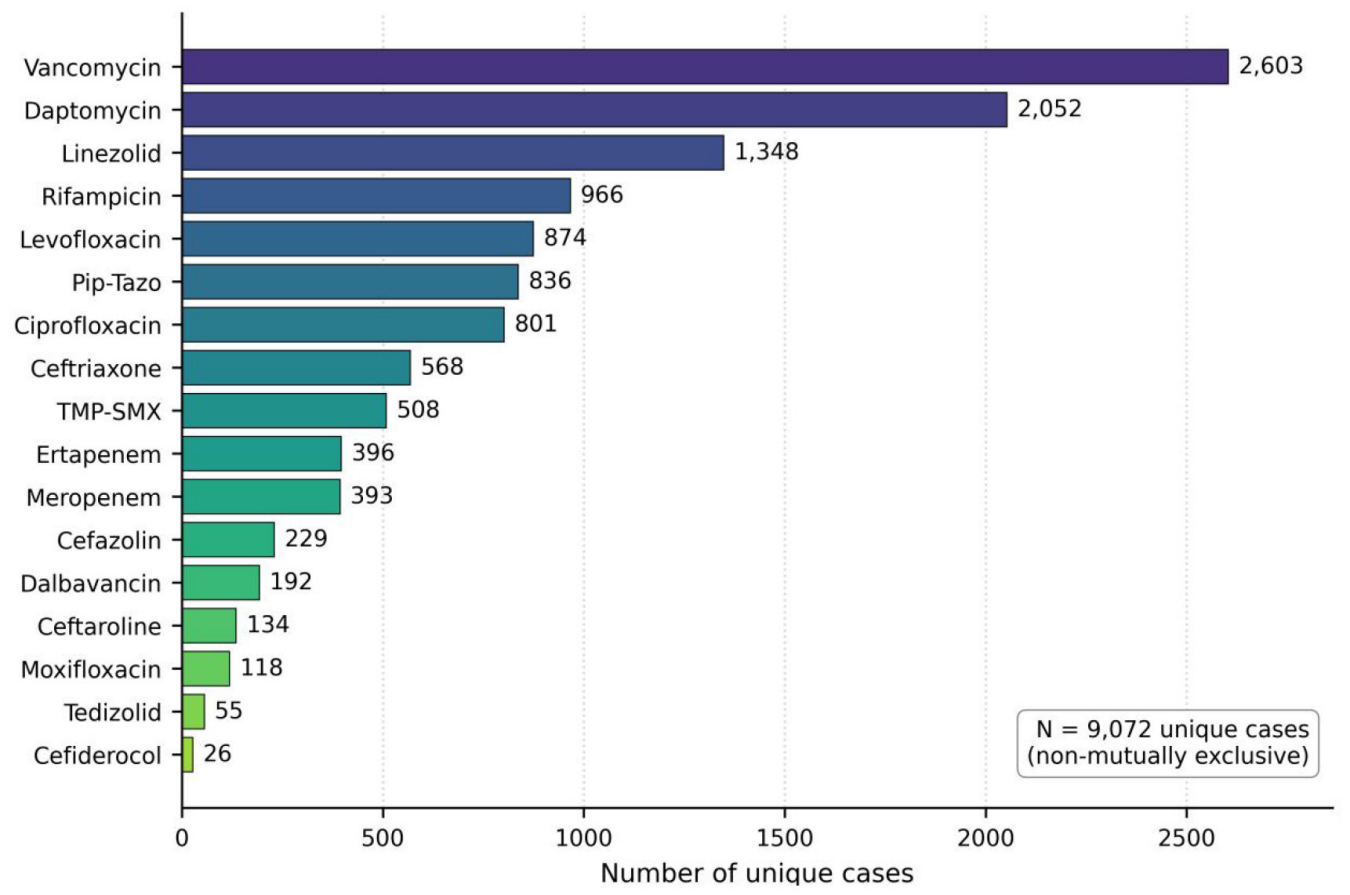
Distribution of target antibiotic reporting frequency in the BJI cohort (unique cases, *N* = 9,072). The labeled bar values sum to 12,099, exceeding the cohort size of 9,072 because a single case may involve more than one target antibiotic (mean approximately 1.33 target antibiotics per case); the figure annotation reports the cohort size. TMP-SMX, trimethoprim-sulfamethoxazole; Pip-Tazo, piperacillin-tazobactam.

**Figure 2:**
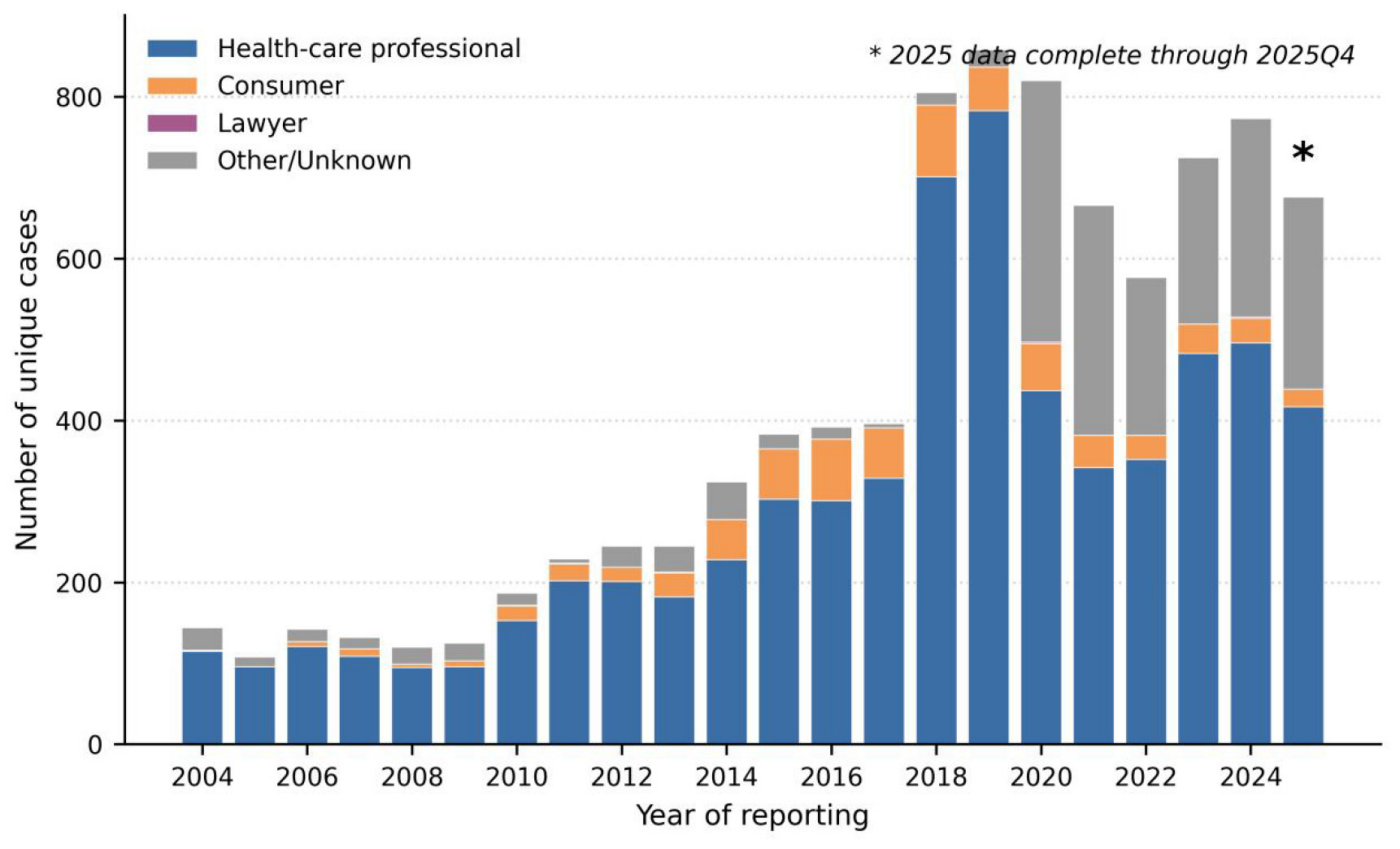
Annual reporting trend of antibiotic-associated adverse drug events in the BJI cohort, 2004–2025, stratified by reporter occupation. Health-care professional (physician, pharmacist, and other licensed clinicians); Consumer = patient or family member; Lawyer = legal/attorney reporter; Other/Unknown = reporter occupation missing or unspecified. Asterisk on the 2025 bar indicates that 2025 data are complete through 2025Q4.

From 20,006,981 deduplicated FAERS cases, 9,072 met the inclusion criteria. The cohort had a median age of 65 years (IQR 54–75); 4,696 (51.8%) were male, 3,077 (33.9%) female, and 1,299 (14.3%) had unknown/missing sex. Serious outcomes (case-level union of death, life-threatening, or hospitalization) were recorded in 5,303 cases (58.5%). Vancomycin was the most frequently reported antibiotic (*n* = 2,603), followed by daptomycin (*n* = 2,052) and linezolid (*n* = 1,348). Table 2 presents demographic characteristics stratified by sex; the per-antibiotic frequencies are visualized in Figure 1 and the annual reporting trend is shown in Figure 2; Supplementary Table S1 summarizes target-antibiotic reporting frequencies.

**Table 2:** Demographic and clinical characteristics of the BJI cohort, stratified by sex.

Characteristic	Overall (*N* = 9,072)	Male (*n* = 4,696)	Female (*n* = 3,077)	Unknown (*n* = 1,299)
Age, median [IQR]	65 [54–75]	65 [54–75]	67 [54–77]	64 [52–75]
< 18 years	159	117	35	7
18-64 years	3,346	1,969	1,167	210
≥65 years	3,912	2,131	1,574	207
Missing	1,655	479	301	875
Death, *n*	623	345	168	110
Life-threatening, *n*	851	477	308	66
Hospitalization, *n*	4,606	2,534	1,565	507
Serious outcome, *n* (%)	5,303 (58.5%)	2,896 (61.7%)	1,793 (58.3%)	614 (47.3%)

Outcome categories are non-mutually exclusive; a single case may have multiple outcome codes. Serious outcome count reflects the case-level union of death, life-threatening, and hospitalization. Age statistics in the ‘Unknown' column refer to cases with missing sex information and available age data; missing-age cases (*n* = 1,655 overall; *n* = 875 in the Unknown-sex column) are excluded from the age summary statistics but counted in the age-stratum rows.

### Disproportionality signals

**Figure 3:**
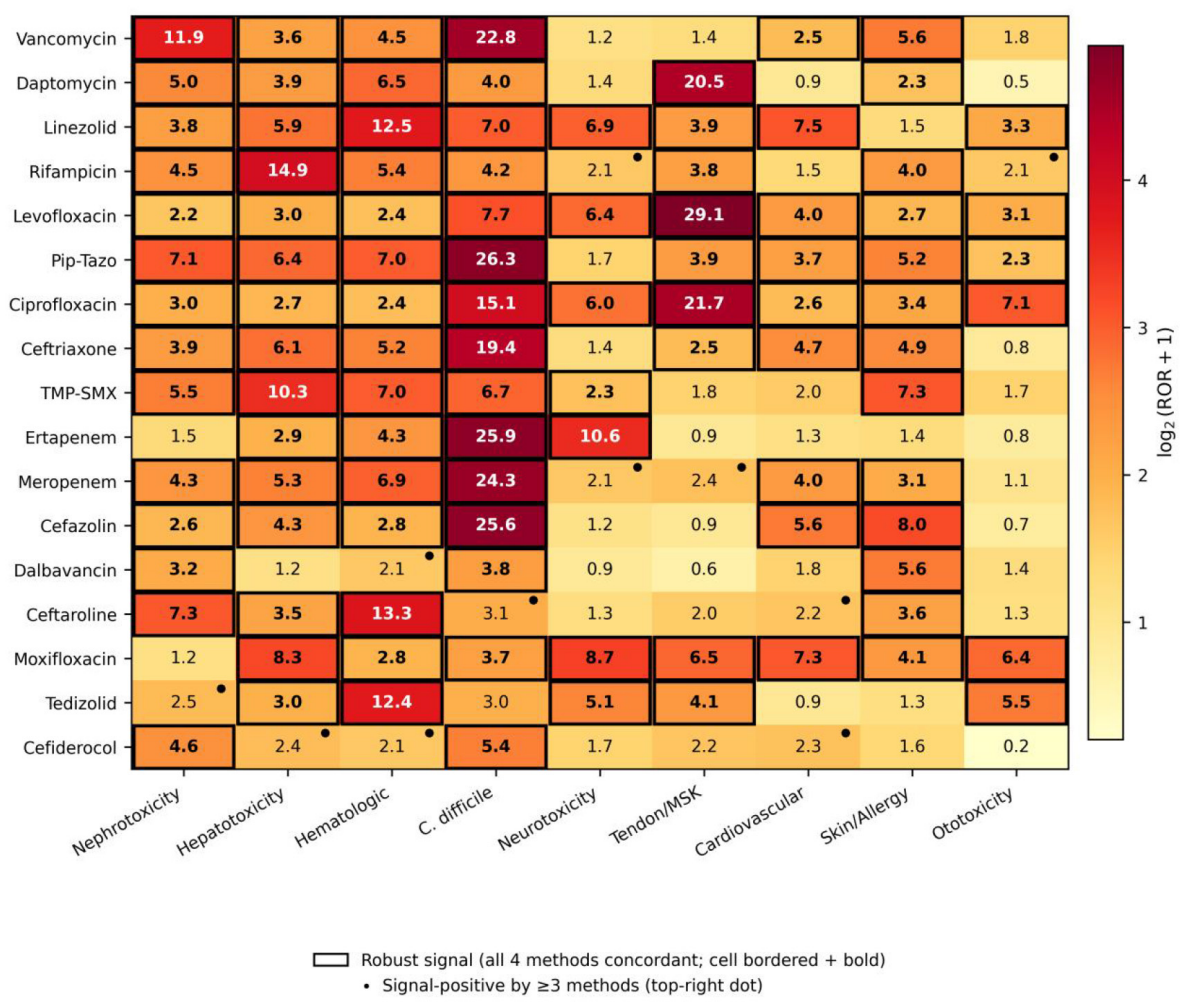
Disproportionality signal heatmap. Annotated values are raw reporting odds ratios (ROR); color intensity corresponds to log_2_ (ROR + 1). Cells with a black border and bold text indicate robust signals where all four methods (ROR, PRR, IC, EBGM) simultaneously meet their predefined thresholds. A small black dot in the upper-right corner of a cell indicates a signal positive by at least three (but not all four) methods.

**Figure 4:**
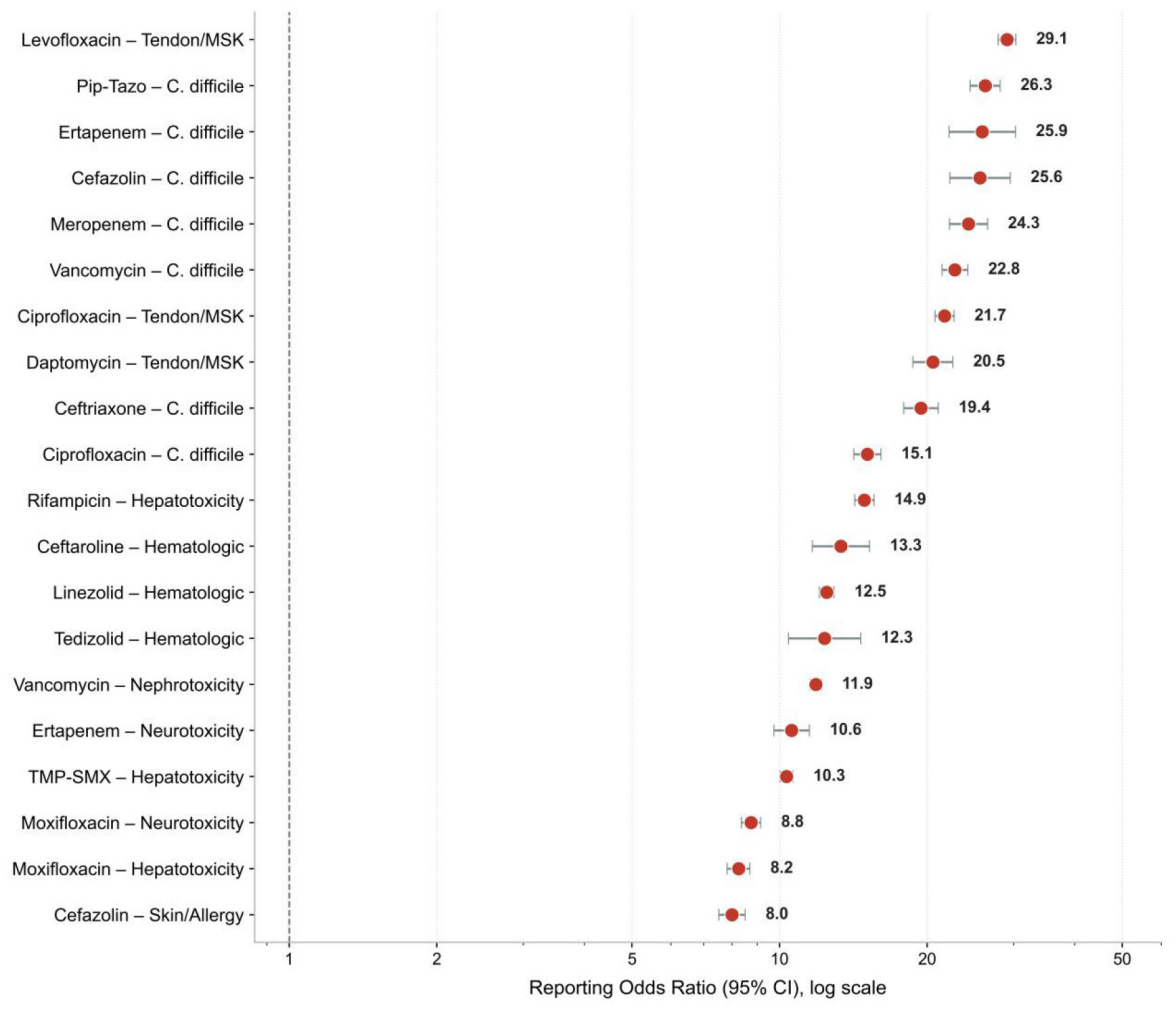
Forest plot of the top 20 drug-ADE signals ranked by ROR with 95% confidence intervals. All signals shown met the robust criterion (all four methods concordant). Reference population, full FAERS database (*N* = 20,006,981).

Of 153 drug-ADE pairs evaluated against the full FAERS database (*N* = 20,006,981), 103 (67.3%) met the robust signal criterion (concordance across all four methods with EB05 ≥2). Case counts in the 2 × 2 tables represent drug-ADE co-occurrence across the entire FAERS database, not limited to the 9,072 BJI cohort. The strongest signals included levofloxacin-tendon/musculoskeletal disorders (ROR = 29.1) and ciprofloxacin-tendon/musculoskeletal disorders (ROR = 21.7) reflecting the fluoroquinolone class effect, broad-spectrum agents-C. difficile infection (piperacillin-tazobactam ROR = 26.3; cefazolin ROR = 25.6), rifampicin-hepatotoxicity (ROR = 14.9), linezolid-hematologic toxicity (ROR = 12.5), and vancomycin-nephrotoxicity (ROR = 11.9). Full results are provided in Supplementary Table S16, with the 17-antibiotic × 9-ADE-category disproportionality matrix visualized in Figure 3 and the top 20 signals by ROR shown in Figure 4. All 103 robust signals remained significant after Benjamini-Hochberg false discovery rate correction (*q* < 0.05).

### Within-cohort contextualization

To contextualize these full-FAERS signals within the BJI cohort, within-cohort disproportionality analysis was performed (*N* = 9,072). Of the 103 full-FAERS robust signals, 28 were retained using the prespecified within-cohort contextualization criteria (ROR lower 95% CI > 1.0 and exposed-event count ≥3) (Supplementary Table S5). Key BJI-retained reporting signals included vancomycin-nephrotoxicity (within-cohort ROR = 3.30, 95% CI 2.95–3.70), linezolid-hematologic toxicity (ROR = 3.65, 95% CI 3.21–4.16), ceftaroline-hematologic toxicity (ROR = 4.81, 95% CI 3.42–6.77), and ertapenem-neurotoxicity (ROR = 4.76, 95% CI 3.50–6.47). Notably, several full-FAERS signals were not replicated within the BJI cohort, including fluoroquinolone-tendon/musculoskeletal disorders (within-cohort a = 0) and vancomycin-C. difficile (within-cohort ROR = 1.29, 95% CI 0.85–1.97). These discrepancies suggest that some full-FAERS signals reflect general prescribing or reporting patterns rather than BJI-specific reporting enrichment, whereas others persist even when compared against the BJI-internal background. In the pre-specified strict drug-linked Tier 1 confirmatory analysis, the four headline BJI-supported signals remained directionally and statistically supported: linezolid-hematologic toxicity (ROR 3.96, 95% CI 3.46–4.53), vancomycin-nephrotoxicity (3.66, 3.24–4.13), ceftaroline-hematologic toxicity (4.63, 3.24–6.62), and ertapenem-neurotoxicity (4.84, 3.54–6.61), with absolute ROR changes no greater than 10.7% (Supplementary Table S3). The remaining BJI-retained signals rested on small exposed-event counts and are interpreted as exploratory, contextual signals; interpretation prioritized the four label-concordant signals (vancomycin-nephrotoxicity, linezolid-hematologic toxicity, ceftaroline-hematologic toxicity, and ertapenem-neurotoxicity) supported by the PS-only and active-comparator analyses.

### Exploratory machine-learning association analysis

**Figure 5:**
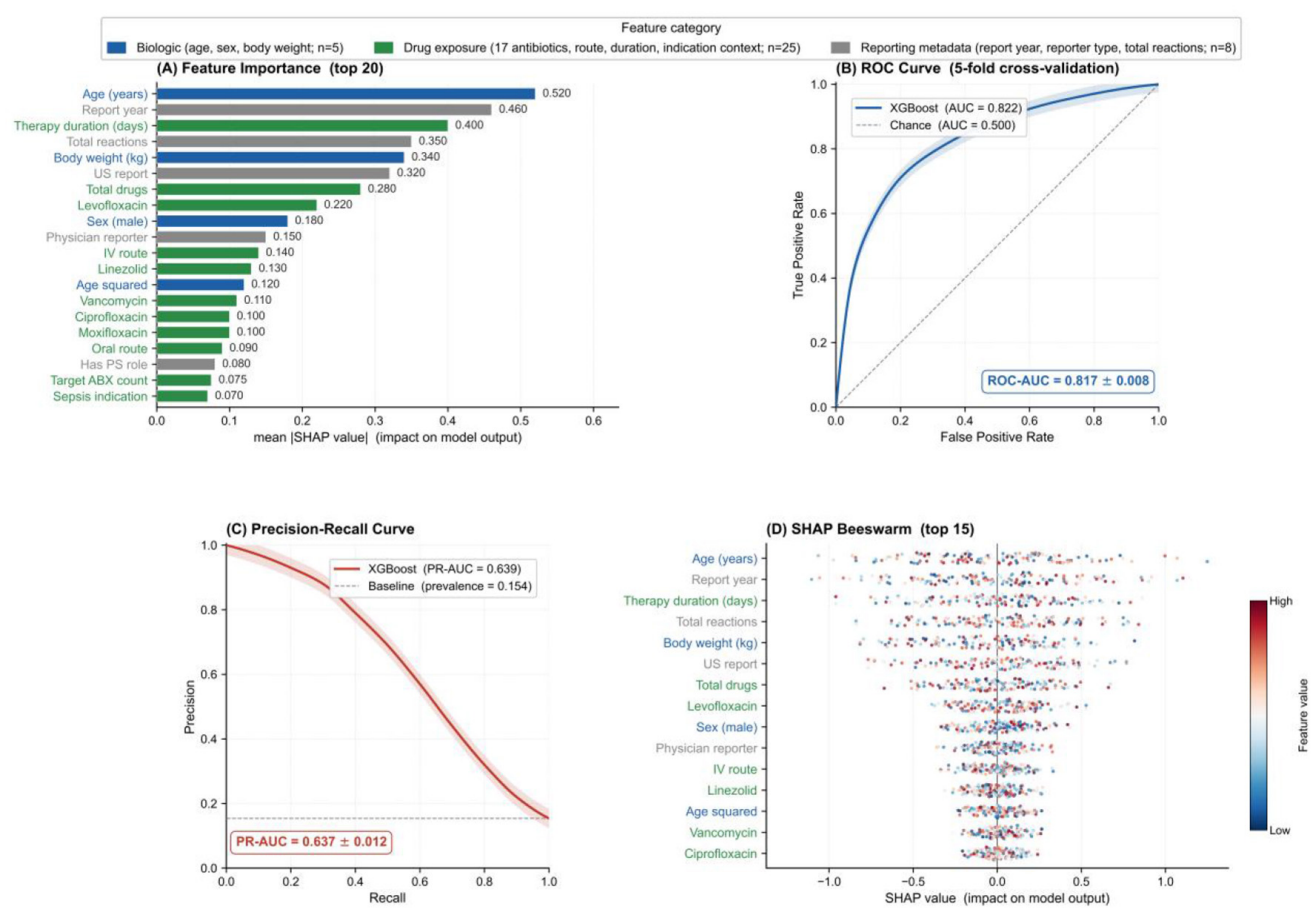
Exploratory XGBoost analysis for severe outcomes (death or life-threatening; *n* = 1,401, 15.4%). The 38 features comprise five biologic (blue), 25 drug-exposure (green), and eight reporting-metadata (gray) variables (Supplementary Table S10). **(A)** Top-20 SHAP feature importance, color-coded by category. **(B)** ROC curve, stratified 5-fold cross-validation (AUC = 0.817 ± 0.008). **(C)** Precision-recall curve (PR-AUC = 0.637 ± 0.012). **(D)** SHAP beeswarm of top-15 features; point color encodes feature value (red = high, blue = low). In panels B and C the curve-legend values (AUC = 0.822; PR-AUC = 0.639) are pooled estimates computed on concatenated cross-validation predictions, whereas the boxed values (ROC-AUC = 0.817 ± 0.008; PR-AUC = 0.637 ± 0.012, reported in the text) are the mean ± SD across the five folds.

The supplementary exploratory XGBoost model achieved a mean ROC-AUC of 0.817 (SD 0.008) and PR-AUC of 0.637 (SD 0.012), with sensitivity 0.666, specificity 0.842, and PPV 0.437. SHAP analysis identified age, report year, therapy duration, total number of reported reactions, and body weight as the features most strongly associated with reported severe outcomes in FAERS (Figure 5A). The ROC curve, precision-recall curve, and SHAP beeswarm are shown in Figures 5B–D Among antibiotic-specific indicators, levofloxacin showed the highest SHAP contribution, followed by linezolid and vancomycin. Non-drug features such as sex and physician reporter type also contributed modestly. Because the model was not developed or calibrated for individual-level clinical prediction, these results should be interpreted as an exploratory association analysis that captures patient characteristics, treatment complexity, and reporting features within FAERS. Although these metadata features carried high weight in the model, they likely reflect report completeness, temporal reporting patterns, and case-coding characteristics rather than direct pathophysiological mechanisms.

### Time-to-onset analysis

**Figure 6:**
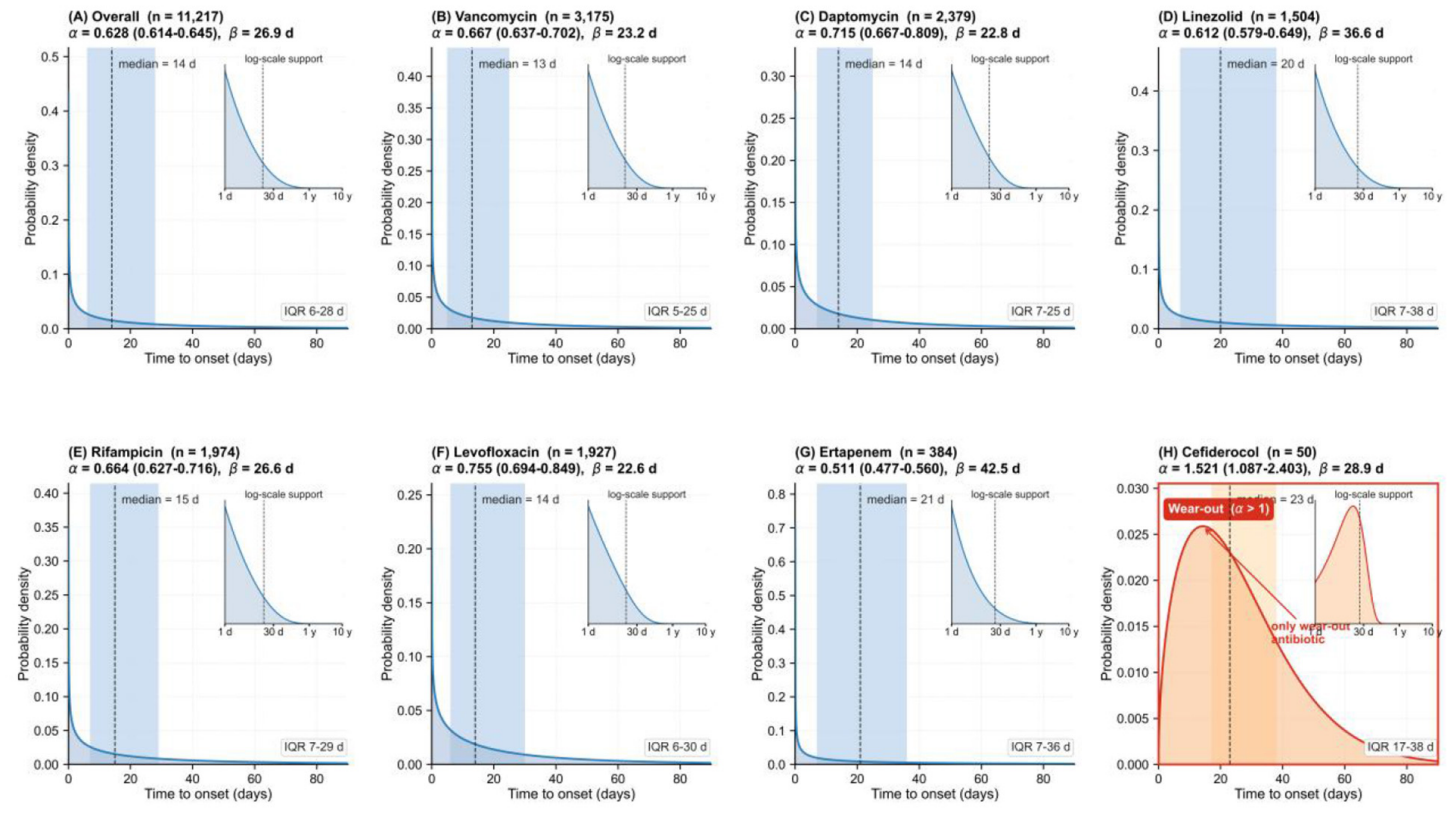
Time-to-onset Weibull fits. Each panel shows fitted Weibull density on a 0–90-day axis with a log-scaled inset to 3,650 days, and reports *n*, α (shape, 95% CI), and β (scale, days); dashed line = median, shaded band = IQR. **(A)** Overall cohort (*n* = 11,217). **(B–G)** Vancomycin, daptomycin, linezolid, rifampicin, levofloxacin, and ertapenem (all early-failure, α < 1; blue). **(H)** Cefiderocol, the only wear-out agent (α > 1; orange with red border). Curves derived from Table 3 parameters.

Time-to-onset (TTO) analysis was based on 11,217 valid drug-therapy records from 4,168 cases with complete date information (45.9% of the cohort; median TTO 14 days; IQR 6–28). The overall cohort exhibited an early-failure Weibull pattern, consistent with reporting patterns concentrated shortly after treatment initiation. Most individual antibiotics also showed α < 1, consistent with early-onset reporting patterns. Cefiderocol was the only agent whose shape parameter confidence interval lay entirely above 1.0 (α = 1.52, 95% CI 1.09–2.40), consistent with a later-onset pattern, though this finding was based on only 50 TTO records and should be interpreted cautiously. Because complete therapy and event dates were available for fewer than half of cases, TTO findings should be interpreted as descriptive reporting patterns rather than incidence-based timing estimates. Table 3 and Figures 6A–G present the overall cohort and six representative antibiotic-specific Weibull parameters; Figure 6H presents the exploratory cefiderocol wear-out pattern. Because date-complete reports were older, more serious, and more often physician-reported than missing-date reports, TTO is therefore interpreted as a documentation-enriched descriptive subset; earliest-case and earliest drug-case sensitivity analyses confirmed that repeated therapy episodes should not be treated as independent patients (Supplementary Table S14). In that conservative strict-linked sensitivity set, the overall early-failure interpretation was retained after earliest-case restriction, whereas drug-specific classifications were based on much smaller linked subsets and were therefore used as sensitivity context rather than replacements for the primary Table 3 estimates. Notably, under the strictest earliest-valid-onset-per-drug-case definition the shape parameter for vancomycin rose above 1 (alpha 1.21, 95% CI 1.08–1.45, wear-out) and ertapenem became indeterminate (alpha 1.13, 95% CI 1.00–1.39); given the small linked subsets (*n* = 180 and 41) and removal of early repeated reports, these drug-specific reversals are interpreted as cautionary sensitivity context rather than evidence against the overall early-failure pattern.

**Table 3:** Weibull time-to-onset parameters. Pip-Tazo = piperacillin-tazobactam.

Drug	*n* ^†^	Median TTO (IQR), days	Shape α (95% CI)	Scale β	Failure type
Overall	11,217	14 (6–28)	0.628 (0.614–0.645)	26.9	Early failure
Vancomycin	3,175	13 (5–25)	0.667 (0.637–0.702)	23.2	Early failure
Daptomycin	2,379	14 (7–25)	0.715 (0.667–0.809)	22.8	Early failure
Linezolid	1,504	20 (7–38)	0.612 (0.579–0.649)	36.6	Early failure
Rifampicin	1,974	15 (7–29)	0.664 (0.627–0.716)	26.6	Early failure
Levofloxacin	1,927	14 (6–30)	0.755 (0.694–0.849)	22.6	Early failure
Pip–Tazo	1,489	11 (5–20)	0.831 (0.756–0.926)	17.4	Early failure
Ciprofloxacin	1,240	18 (7–36)	0.615 (0.583–0.666)	33.3	Early failure
Ceftriaxone	752	14 (5–25)	0.594 (0.557–0.642)	25.5	Early failure
TMP–SMX	931	15 (6–31)	0.700 (0.648–0.780)	25.3	Early failure
Ertapenem	384	21 (7–36)	0.511 (0.477–0.560)	42.5	Early failure
Meropenem	553	18 (7–38)	0.671 (0.615–0.749)	31.7	Early failure
Cefazolin	399	9 (3–19)	0.617 (0.542–0.835)	15.7	Early failure
Dalbavancin	161	25 (10–52)	0.812 (0.710–1.012)	39.9	Indeterminate
Ceftaroline	74	24 (13–38)	0.840 (0.704–1.531)	37.4	Indeterminate
Moxifloxacin	74	36 (10–83)	0.687 (0.584–0.883)	61.7	Early failure
Tedizolid	64	42 (17–64)	0.506 (0.451–0.662)	86.0	Early failure
Cefiderocol	50	23 (17–38)	1.521 (1.087–2.403)	28.9	Wear-out

^†^Drug-specific rows represent drug-therapy episodes linked via primaryid. Because a single case may involve multiple target antibiotics, each case may contribute to more than one drug-specific row. The sum of drug-specific counts therefore exceeds the overall unique record count (11,217). Because a single case may contribute multiple therapy episodes for the same antibiotic (separate THER start dates linked via primaryid and drug_seq), each drug-specific n counts therapy episodes rather than unique patients and may exceed that antibiotic's total cohort case count in Supplementary Table S1.

BP cohort (*n* = 3,581) was constructed from the deduplicated FAERS INDI table using 26 bacterial-pneumonia MedDRA preferred terms (excluding COVID-19, Pneumocystis jirovecii, viral, fungal, organizing, hypersensitivity, idiopathic interstitial, radiation, community-acquired, and eosinophilic variants). Within-BP-cohort 2 × 2 contingency tables used the same ADE preferred-term pools (Supplementary Table S4) and the same Haldane-Anscombe continuity correction (0.5 added to all four cells when any cell count was zero) as the main BJI within-cohort analysis. An infective-endocarditis (IE) long-course comparator cohort (*N* = 1,500) was constructed identically from 26 endocarditis-spectrum MedDRA preferred terms (non-infective variants excluded), using the same continuity-corrected within-cohort ROR estimator and ADE preferred-term pools (Supplementary Table S9). † Ertapenem-IE was sparse (*n* = 34 IE-exposed cases; exposed events a = 1) and is not interpretable. Ratios are computed from unrounded ROR estimates.

Per-case primary role of vancomycin assigned by hierarchy PS > SS > C > I when a case had multiple vancomycin records. ‘CDI/related as vancomycin's indication' denotes at least one vancomycin record linked via indi_drug_seq to a CDI-spectrum preferred term (Clostridium difficile infection, Clostridium difficile colitis, Pseudomembranous colitis, or Clostridioides difficile infection). The substantial non-PS proportion (81.5%) together with the explicit CDI-as-indication proportion (34.0%) indicate that the vast majority of vancomycin-CDI co-occurrence in FAERS reflects vancomycin being used to treat CDI rather than being suspected of causing it.

### Sensitivity analysis

In the primary-suspect-only (PS-only) within-cohort sensitivity analysis (*n* = 7,389 cases), key signals showed partial consistency with the main analysis. Signals that remained robust included vancomycin-nephrotoxicity (ROR = 3.85, 95% CI 3.38–4.40) and linezolid-hematologic toxicity (ROR = 3.87, 95% CI 3.35–4.47). In contrast, several high-ranking full-FAERS signals, including fluoroquinolone-tendon/musculoskeletal disorders and broad-spectrum antibiotic-C. difficile associations, were attenuated or based on sparse PS counts. For pairs with PS-only a = 0, continuity-corrected ROR estimates are unstable and should not be interpreted as PS-only evidence of a signal. Full PS-only results for the top 20 pairs are provided in Supplementary Table S15. Drug-role attribution supported the four headline signals: PS-only exposed-event counts were 546 for vancomycin-nephrotoxicity, 386 for linezolid-hematologic toxicity, 42 for ceftaroline-hematologic toxicity, and 50 for ertapenem-neurotoxicity, with PS-only RORs remaining above unity for all four (Supplementary Table S7).

Exploratory active-comparator analyses within mutually exclusive BJI-exposed cases provided additional context for indication and class effects. Vancomycin showed higher nephrotoxicity reporting than daptomycin (active-comparator OR/ROR 4.06, 95% CI 3.42–4.83), daptomycin showed higher myotoxicity reporting than vancomycin (24.64, 15.61–38.89), linezolid showed higher hematologic-toxicity reporting than vancomycin (3.05, 2.60–3.59), ceftaroline showed higher hematologic-toxicity reporting than ceftriaxone (4.79, 3.19–7.20), and ertapenem showed higher neurotoxicity reporting than meropenem (5.96, 2.98–11.91). These comparisons are exploratory and uncontrolled for the confounders discussed in the Limitations (Supplementary Table S8).

### External validation against bacterial-pneumonia comparator

**Figure 7:**
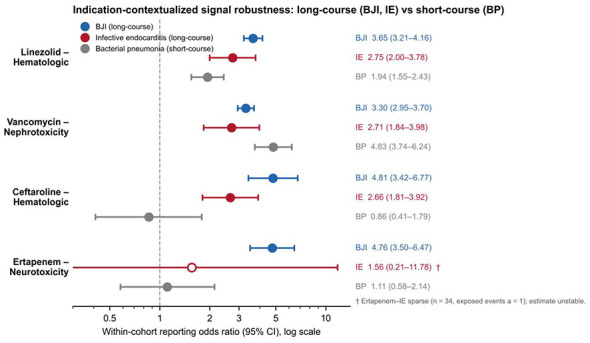
Indication-contextualized signal robustness across long-course (BJI, infective endocarditis) vs. short-course (bacterial pneumonia) infections. Within-cohort reporting odds ratios (ROR) with 95% confidence intervals for the four prioritized antibiotic-ADE signals in each cohort; the dashed line marks ROR = 1. Linezolid- and ceftaroline-hematologic signals remain elevated in both long-course cohorts (BJI, IE) but attenuate in short-course BP (duration-dependent), whereas vancomycin-nephrotoxicity is comparable across indications (class/acute-dose effect). ^†^ Ertapenem-IE is sparse (n = 34, exposed events a = 1) and not interpretable.

To assess whether the four prioritized BJI-supported reporting signals (vancomycin-nephrotoxicity, linezolid-hematologic, ceftaroline-hematologic, ertapenem-neurotoxicity) reflect indication-contextualized reporting enrichment vs. broader class effects, we compared within-cohort ROR estimates against a bacterial-pneumonia (BP) comparator cohort (*n* = 3,581; Table 1 and Figure 7). Three signals exhibited substantially higher ROR in BJI than in BP, namely linezolid-hematologic (BJI 3.65, 95% CI 3.21–4.16 vs BP 1.94, 1.55–2.43; BJI/BP ratio 1.88), ceftaroline-hematologic (BJI 4.81, 3.42–6.77 vs BP 0.86, 0.41–1.79; ratio 5.59), and ertapenem-neurotoxicity (BJI 4.76, 3.50–6.47 vs BP 1.11, 0.58–2.14; ratio 4.26), consistent with cumulative-exposure-dependent toxicities amplified by the prolonged BJI treatment course. In contrast, vancomycin-nephrotoxicity was comparable or slightly more pronounced in BP (BJI 3.30, 2.95–3.70 vs. BP 4.83, 3.74–6.24; ratio 0.68), consistent with this signal representing a class-effect, acute-dose-related toxicity rather than a duration-dependent one.

Figure 7 Indication-contextualized signal robustness across long-course (BJI, infective endocarditis) vs. short-course (bacterial pneumonia) infections. Within-cohort reporting odds ratios (ROR) with 95% confidence intervals for the four prioritized antibiotic-ADE signals in each cohort; the dashed line marks ROR = 1. Linezolid- and ceftaroline-hematologic signals remain elevated in both long-course cohorts (BJI, IE) but attenuate in short-course BP (duration-dependent), whereas vancomycin-nephrotoxicity is comparable across indications (class/acute-dose effect). † Ertapenem-IE is sparse (*n* = 34, exposed events a = 1) and not interpretable.

## Discussion

This indication-enriched pharmacovigilance analysis evaluated antibiotic safety signals among 9,072 BJI cases against the full FAERS database (*N* = 20,006,981) and identified 103 of 153 evaluated drug-ADE pairs as concordant reporting signals satisfying a prespecified four-method robust criterion, of which 28 retained directional support after BJI within-cohort contextualization. The central contribution is therefore not the rediscovery of well-known antibiotic toxicities, but the separation of broad FAERS reporting enrichment from signals that remain supported in an indication-enriched BJI background. The integration of full-database signal detection, BJI-internal contextualization, PS-only sensitivity analysis, exploratory machine learning, and time-to-onset characterization provides a transparent framework for prioritizing safety hypotheses during prolonged antibiotic therapy.

The strong nephrotoxicity signal for vancomycin (ROR = 11.9) aligns with established clinical evidence on dose-dependent renal injury during prolonged therapy (5). The fluoroquinolone-tendon/musculoskeletal signals (levofloxacin ROR = 29.1; ciprofloxacin ROR = 21.7) are consistent with known class effects (7). Linezolid-hematologic toxicity (ROR = 12.5) corresponds to the well-described duration-dependent thrombocytopenia risk (6). The prominent C. difficile signals across multiple broad-spectrum agents reflect the established association between antibiotic exposure and Clostridioides difficile infection (CDI) risk (Deshpande et al.(24); the original 2013 publication used the prior nomenclature Clostridium difficile, which was reclassified to Clostridioides in 2016) (25). However, not all established full-FAERS signals were reproduced in the BJI-internal analysis (see Within-Cohort Contextualization), indicating that these findings should be interpreted as background pharmacovigilance context rather than BJI-specific reporting enrichment.

Ceftaroline-hematologic toxicity was supported by within-cohort analysis (BJI-supported) and warrants further evaluation, consistent with prior reports of neutropenia during extended ceftaroline therapy (26, 27). Daptomycin-tendon/musculoskeletal reporting was observed in the full-FAERS analysis (ROR = 20.5) but the within-cohort exposed-event count for the operationally restricted Tendon/MSK preferred-term pool was zero (Supplementary Table S5). Preferred-term-level stratification of the 2,052 daptomycin BJI cases (Supplementary Table S6) shows that myotoxicity-related events were in fact reported in 330 unique cases (16.1%), predominantly as BLOOD CREATINE PHOSPHOKINASE INCREASED (*n* = 173) and RHABDOMYOLYSIS (*n* = 121); the apparent within-cohort a = 0 reflects the operational restriction described in Methods (RHABDOMYOLYSIS interpreted separately) rather than absence of biological signal. The proportion of daptomycin cases with any myotoxicity preferred term in the BJI cohort (16.1%) exceeds that in the full-FAERS daptomycin population (9.1%), consistent with the longer mean therapy course typical of BJI management and reinforcing routine creatine-kinase monitoring during prolonged daptomycin therapy as recommended by current product labelling (28). When evaluated as a separate myotoxicity construct, daptomycin was associated with 330 myotoxicity-related BJI cases (16.1% of 2,052 daptomycin-exposed BJI cases) and a within-BJI ROR of 7.45 (95% CI 6.16–9.02).

The Weibull TTO analysis indicated a predominantly early-onset reporting pattern in this BJI cohort (overall α = 0.628; median 14 days, IQR 6-28), supporting an emphasis on early-treatment monitoring. The cefiderocol later-onset pattern (Table 3; α = 1.52, 95% CI 1.09–2.40; Figure 6H) is biologically plausible given its use in difficult-to-treat infections that may require extended courses, but four alternative explanations should be weighed before accepting it as a drug-intrinsic wear-out signature. First, the estimate is statistically fragile with only 50 TTO records. Second, cefiderocol was first approved in 2019 and its post-market reporting is still maturing, so a wear-out shape may partly reflect delayed reporting cadence rather than a true delayed-onset toxicity. Third, cefiderocol is typically reserved for carbapenem-resistant Gram-negative infections that require extended therapy (commonly 4–6 weeks or longer) (29), so the later-onset reporting pattern may partly reflect the longer mean therapy duration intrinsic to its indication rather than a cefiderocol-specific timing of toxicity. Fourth, in BJI cefiderocol is often used as salvage therapy after earlier-line antibiotic failure; the wear-out shape may therefore partly reflect carry-over effects from prior antibiotic exposure (channeling or protopathic bias) rather than direct cefiderocol causation. Pending larger post-marketing series with controlled adjudication of antibiotic timing and indication, this signal should be treated as exploratory. In addition, cefiderocol's siderophore cephalosporin structure raises a biologically plausible, but unproven, hypothesis that prolonged exposure or iron-transport-related pharmacologic mechanisms could contribute to delayed reporting patterns in selected patients (29). However, because FAERS cannot evaluate iron metabolism or other mechanistic and laboratory variables, this mechanism remains hypothesis-generating and requires dedicated clinical and mechanistic validation.

The PS-only sensitivity analysis revealed important nuances. Several strong full-FAERS signals were substantially attenuated or absent when restricted to primary suspect records, including fluoroquinolone-tendon/musculoskeletal disorders (PS-only a = 0) and broad-spectrum antibiotic-C. difficile associations (e.g., vancomycin PS-only ROR = 0.89 vs. full-FAERS ROR = 22.78). Stratification of the 2,215 full-FAERS vancomycin-CDI co-occurrence cases by vancomycin's drug role and indication (Table 4) showed that vancomycin was coded as concomitant in 49.0% and as secondary suspect in 31.9% of these cases (only 18.5% as primary suspect), and that 34.0% of cases explicitly listed CDI or a CDI-spectrum term as vancomycin's indication. In other words, oral vancomycin is a guideline-recommended therapy for CDI, and in the majority of vancomycin-CDI co-occurrence reports vancomycin is present because it is being used to treat the CDI rather than being suspected of causing it. This is a textbook manifestation of indication vs. suspect ambiguity (sometimes termed ‘protopathic bias') in spontaneous reporting systems and methodologically explains the dramatic reversal of the vancomycin-CDI signal between unrestricted and PS-only analyses. By contrast, signals such as linezolid-hematologic toxicity and vancomycin-nephrotoxicity remained consistent in PS-only analysis, supporting their robustness because the drugs involved are not used to treat the very events being analyzed. These findings underscore the importance of interpreting FAERS disproportionality signals in the context of drug role coding and indication-suspect attribution, and highlight that not all “robust” full-FAERS signals carry equal evidentiary weight for drug-specific safety concerns. A parallel phenomenon is observed for ceftriaxone-tendon/musculoskeletal (full-FAERS ROR = 2.54; within-cohort a = 0; Supplementary Table S5), where the absence of a BJI within-cohort signal likely reflects the same indication vs. suspect dynamics rather than absence of biological effect.

**Table 4:** Vancomycin drug-role × CDI-as-indication cross-tabulation in 2,215 full-FAERS vancomycin-CDI co-occurrence cases.

Vancomycin drug role	No CDI/related as indication	CDI/related as vancomycin's indication	Total
Primary suspect (PS)	198 (8.9%)	212 (9.6%)	410 (18.5%)
Secondary suspect (SS)	397 (17.9%)	310 (14.0%)	707 (31.9%)
Concomitant (C)	857 (38.7%)	228 (10.3%)	1,085 (49.0%)
Interacting (I)	11 (0.5%)	2 (0.1%)	13 (0.6%)
Total	1,463 (66.0%)	752 (34.0%)	2,215 (100%)

The table documents the indication vs. suspect ambiguity (protopathic bias) underlying the dramatic vancomycin-CDI signal reversal between the full-FAERS analysis (ROR 22.78, all roles pooled) and the primary-suspect-only analysis (ROR 0.89; Supplementary Table S15).

The fluoroquinolone tendon-toxicity class effect is well established; its attenuation in the BJI within-cohort and PS-only analyses likely reflects indication- and attribution-specific reporting dynamics in BJI. Fluoroquinolones are commonly used as combination, step-down, or suppressive therapy in BJI, and may therefore be coded as secondary rather than primary suspect drugs. In parallel, tendon and musculoskeletal events in BJI patients may be clinically attributed to postoperative recovery, immobilization, underlying orthopedic disease, or infection-related disability. Thus, the full-FAERS signal reflects a known class toxicity, whereas the attenuated BJI/PS-only signal suggests weaker drug-specific attribution within this complex indication context.

The external comparison against a bacterial-pneumonia (BP) cohort (*n* = 3,581; Table 3) supports a refined interpretation that separates duration-dependent from class-effect toxicities. The three BJI-supported signals involving linezolid-hematologic, ceftaroline-hematologic, and ertapenem-neurotoxicity showed BJI ROR substantially higher than BP ROR (ratios 1.88, 5.59, and 4.26 respectively), consistent with the established cumulative-exposure dependence of linezolid- and oxazolidinone-related bone-marrow suppression, ceftaroline-related neutropenia with ≥7-day therapy, and ertapenem-related neurotoxicity in prolonged regimens. In contrast, vancomycin-nephrotoxicity was comparable or slightly higher in the BP cohort (BJI/BP ratio 0.68), which is biologically plausible because vancomycin-associated acute kidney injury is principally driven by trough exposure and concurrent nephrotoxin burden rather than total treatment duration, and BP/critical-care populations frequently exhibit higher baseline AKI risk. The comparator analysis therefore differentiates duration-amplified BJI-specific signals from class-effect signals whose prominence is shared across alternative indication-contextualized backgrounds, an interpretation not derivable from BJI-internal analysis alone. The bacterial-pneumonia comparator represents a partial short-course antibiotic-exposed comparator only and cannot fully remove indication bias because pneumonia reports may involve ICU illness, acute kidney injury, mechanical ventilation, and nephrotoxin exposure, whereas BJI reports may reflect longer therapy and hardware-associated complexity. A second long-course indication, an infective-endocarditis (IE) comparator (*n* = 1,500; Figure 7, Supplementary Table S9), provided further contrast. Linezolid-hematologic (IE ROR 2.75, 95% CI 2.00–3.78) and ceftaroline-hematologic (2.66, 1.81–3.92) signals remained elevated in IE as in BJI, both long-course infections, whereas they were attenuated in short-course pneumonia, reinforcing a duration-dependent interpretation. Vancomycin-nephrotoxicity was comparable across BJI, IE and BP (IE 2.71, 1.84–3.98), consistent with a class/acute-dose effect. The ertapenem-IE estimate was sparse (exposed events a = 1) and was not interpretable.

The observed discriminative performance (ROC-AUC = 0.817) likely reflects the more specific severe-outcome endpoint (death or life-threatening events, 15.4% prevalence) and the inclusion of reporting-complexity variables. However, variables such as reaction count, therapy duration, and report year may partially capture reporting thoroughness, reporting-era effects, or case complexity rather than true biological risk. The machine-learning component should therefore be interpreted as a supplementary association analysis and feature-prioritization exercise, not as a deployable clinical prediction model. Critically, of the top 20 SHAP features (Figure 5A), five are reporting-metadata variables (Report year, Total reactions, US report, Physician reporter, Has PS role for target antibiotic; gray bars) that capture reporting behavior and case-coding characteristics rather than biological risk; these features should not be interpreted causally. The machine-learning component therefore serves as an exploratory feature-prioritization exercise rather than evidence that, for example, calendar year or reaction-count proxies are biological determinants of severe outcomes.

## Strengths

First, the 22-year analytic window and inclusion of 17 BJI-relevant antibiotics provide breadth and statistical power uncommon in single-agent pharmacovigilance studies. Second, the prespecified four-method robust signal definition (ROR, PRR, IC, EBGM) with concordant thresholds reduces reliance on any single disproportionality measure and limits chance-driven findings. Third, the explicit indication-enriched within-cohort contextualization separates broad FAERS reporting enrichment from signals that persist in a BJI-specific background, directly addressing confounding by indication. Fourth, the PS-only sensitivity analysis exposes signals driven mainly by secondary-suspect or concomitant reporting and prevents over-interpretation of full-FAERS signals as drug-specific risks. Fifth, the analysis follows the READUS-PV reporting framework and the complete analytic pipeline, indication dictionary, and ADE category mappings will be publicly shared at the time of publication, supporting reproducibility.

## Limitations

First, FAERS is subject to reporting biases including substantial under-reporting of adverse drug reactions, stimulated reporting, and notoriety bias (30). Second, the BJI cohort was defined using INDI table terms that include both implant-associated and non-implant bone and joint infections, and Tier 2 contextual criteria may have included a minority of non-BJI cases, although Tier 2 contributed only 1.4% of the cohort. The pre-specified strict drug-linked Tier 1 confirmatory analysis (*N* = 8,309; 763 cases excluded for lack of explicit drug-linkage, a larger exclusion than the 128 contextual Tier 2 cases) supported all four BJI-supported signals (Supplementary Table S3). Third, disproportionality was evaluated against the full FAERS database; the identified signals reflect overall reporting enrichment and should not be interpreted as BJI-specific risk estimates. Although a bacterial-pneumonia (BP) comparator cohort was analyzed to externally validate four signals (Table 3), BP represents only one of several possible indication-specific comparators (e.g., complicated skin and soft-tissue infection, infective endocarditis), and expanded comparator analyses across additional long- and short-course indications are warranted. Fourth, the exploratory machine learning analysis primarily recapitulated known clinical and reporting associations and does not constitute a calibrated or validated risk prediction tool. Fifth, TTO analysis was limited to cases with complete date information, and cases with complete dates may differ systematically from those with missing timing data. Sixth, the 2004–2025 study period spans changes in reporting practices, drug availability, clinical guidelines, and coding behavior, introducing temporal heterogeneity that could not be fully controlled. Seventh, indication confounding by the underlying infection severity, surgical stress, and concomitant medications could not be formally controlled in this observational pharmacovigilance design(31). More generally, FAERS cannot estimate incidence, absolute risk, causality, dose-response relationships, renal-function effects, laboratory-confirmed toxicity, or the adjudicated culprit drug in multi-antibiotic regimens; these limitations are inherent to spontaneous-report data. An infective-endocarditis long-course comparator was analyzed (Figure 7; Supplementary Table S9), and comparisons across further indications (e.g., complicated skin and soft-tissue infection) remain warranted.

### Clinical implications

Our four BJI-supported safety signals (vancomycin-nephrotoxicity, linezolid-hematologic toxicity, ceftaroline-hematologic toxicity, ertapenem-neurotoxicity) are largely consistent with existing product labeling and IDSA-endorsed monitoring guidance for prolonged antibiotic therapy, including AUC-guided trough monitoring with regular serum creatinine surveillance for vancomycin (5), weekly complete blood counts for linezolid during prolonged therapy, reflecting its well-described duration-dependent thrombocytopenia risk (6), creatine-kinase surveillance for daptomycin, and dose adjustment with neurological status assessment for ertapenem in older adults or those with renal impairment. The added value of this indication-contextualized analysis is therefore twofold. First, it provides empirical support that these monitoring priorities (derived largely from shorter-course or general-population evidence) remain salient in the prolonged-therapy BJI setting and are not eclipsed by other competing signals (Table 3 duration vs. class analysis). Second, it surfaces a single incremental clinical priority, namely consideration of weekly complete blood counts during ceftaroline regimens of seven days or longer, where prior evidence has been less extensive than for vancomycin or linezolid (26, 27) and where the BJI within-cohort ROR for hematologic toxicity (4.81) is the highest among the four BJI-supported signals. These monitoring implications should be regarded as hypothesis-generating from spontaneous reporting data and prospectively validated before incorporation into formal BJI antibiotic stewardship protocols. The principal findings therefore represent label-concordant monitoring priorities that remained prominent in the BJI reporting context rather than novel toxicities: vancomycin-nephrotoxicity and linezolid-hematologic toxicity align with established monitoring guidance, ceftaroline-hematologic toxicity is consistent with prolonged-exposure hematologic reports, ertapenem-neurotoxicity is concordant with label-described CNS events, and daptomycin-myotoxicity aligns with creatine-kinase/rhabdomyolysis monitoring (Supplementary Table S17).

Ertapenem also warrants specific attention. Although ertapenem is generally considered well tolerated and is often viewed as having a comparatively favorable neurotoxicity profile among carbapenems, the BJI-contextualized signal for neurotoxicity remained prominent in this prolonged-treatment setting. This finding is consistent with known carbapenem-associated central nervous system adverse events, including seizures and encephalopathy (32), but suggests that neurologic monitoring may deserve particular attention during extended BJI treatment courses, especially in older adults and patients with renal impairment (33).

## Conclusions

In this multi-method, indication-contextualized FAERS pharmacovigilance analysis of 9,072 BJI cases, 103 of 153 antibiotic-ADE pairs met the prespecified full-FAERS robust signal criterion, and 28 were retained after BJI within-cohort contextualization. Vancomycin-nephrotoxicity, linezolid-hematologic toxicity, ceftaroline-hematologic toxicity, and ertapenem-neurotoxicity were the most consistently supported BJI-internal signals. Weibull modeling suggested that most reports occurred early after treatment initiation, whereas cefiderocol showed an exploratory wear-out pattern requiring further validation. Overall, this framework helps distinguish BJI-supported signals from broader FAERS reporting enrichment and supports prioritization of safety monitoring during prolonged BJI antibiotic therapy. All findings remain hypothesis-generating and require validation in EHR, registry, or prospective cohorts before informing treatment recommendations.

## Data Availability

The original contributions presented in the study are included in the article/supplementary material, further inquiries can be directed to the corresponding author.
