# Supplementary material for: Antibiotic-associated adverse events in bone and joint infections: a FAERS pharmacovigilance study
Source: Front Med (Lausanne). 2026 Jul 24;13:1887555. doi: 10.3389/fmed.2026.1887555 (PMC13449803; doi:10.3389/fmed.2026.1887555)
Supplement: Supplementary file 1 [file Supplementary_file_1.docx]

# Supplementary Material

## Table S1. Target Antibiotics and Reporting Frequencies in the BJI Cohort

| **Antibiotic** | **Primary search term / active ingredient** | **BJI cohort cases, n** |
| --- | --- | --- |
| Vancomycin | vancomycin | 2,603 |
| Daptomycin | daptomycin | 2,052 |
| Linezolid | linezolid | 1,348 |
| Rifampicin | rifampicin/rifampin | 966 |
| Levofloxacin | levofloxacin | 874 |
| Piperacillin-tazobactam | piperacillin-tazobactam / piperacillin tazobactam | 836 |
| Ciprofloxacin | ciprofloxacin | 801 |
| Ceftriaxone | ceftriaxone | 568 |
| Trimethoprim-sulfamethoxazole | trimethoprim-sulfamethoxazole / TMP-SMX | 508 |
| Ertapenem | ertapenem | 396 |
| Meropenem | meropenem | 393 |
| Cefazolin | cefazolin | 229 |
| Dalbavancin | dalbavancin | 192 |
| Ceftaroline | ceftaroline | 134 |
| Moxifloxacin | moxifloxacin | 118 |
| Tedizolid | tedizolid | 55 |
| Cefiderocol | cefiderocol | 26 |

Note — Frequencies are case-level counts of target antibiotic reports among the 9,072 BJI cohort cases. Counts are non-mutually exclusive because a single case may include more than one target antibiotic.

## Table S2. BJI Cohort Qualifying Indication Dictionary

| MedDRA PT | Tier/Role | Frequency |
| --- | --- | --- |
| OSTEOMYELITIS | Tier 1 | 7637 |
| DEVICE RELATED INFECTION | Tier 1 | 4406 |
| INTERVERTEBRAL DISCITIS | Tier 1 | 1580 |
| POST PROCEDURAL INFECTION | Tier 1 | 1155 |
| SEPTIC ARTHRITIS | Tier 1 | 227 |
| IMPLANT SITE INFECTION | Tier 1 | 103 |
| MEDICAL DEVICE SITE JOINT INFECTION | Tier 1 | 263 |
| DEVICE RELATED SEPSIS | Tier 1 | 200 |
| JOINT INFECTION | Tier 1 | 271 |
| BONE INFECTION | Tier 1 | 14 |
| ARTHRITIS INFECTIVE | Tier 1 | 415 |
| OSTEOMYELITIS BACTERIAL | Tier 1 | 305 |
| OSTEOMYELITIS CHRONIC | Tier 1 | 239 |
| OSTEOMYELITIS ACUTE | Tier 1 | 113 |
| MEDICAL DEVICE SITE INFECTION | Tier 1 | 83 |
| MEDICAL DEVICE SITE ABSCESS | Tier 1 | 22 |
| OSTEOMYELITIS SALMONELLA | Tier 1 | 12 |
| PROSTHETIC JOINT INFECTION | Tier 1 | 0 (zero matches) |
| STAPHYLOCOCCAL BONE INFECTION | Tier 1 | 0 (zero matches) |
| ORTHOPAEDIC DEVICE INFECTION | Tier 1 | 0 (zero matches) |
| STAPHYLOCOCCAL INFECTION | Tier 2 (generic infection) | 1092 |
| INFECTION | Tier 2 (generic infection) | 216 |
| BACTERIAL INFECTION | Tier 2 (generic infection) | 72 |
| CELLULITIS | Tier 2 (generic infection) | 157 |
| ABSCESS | Tier 2 (generic infection) | 36 |
| WOUND INFECTION | Tier 2 (generic infection) | 39 |
| SKIN INFECTION | Tier 2 (generic infection) | 46 |
| ARTHROPLASTY | Tier 2 (ortho procedure context) | 103 |
| FRACTURE | Tier 2 (ortho procedure context) | 144 |
| SPINAL FUSION | Tier 2 (ortho procedure context) | 11 |
| ORTHOPAEDIC PROCEDURE | Tier 2 (ortho procedure context) | 4 |
| ORTHOPEDIC | Tier 2 (ortho procedure context) | 1 |

Note — Frequencies represent case-level counts of target-antibiotic-linked qualifying indications. A single case may contribute to multiple indication terms (non-mutually exclusive). Zero-match terms are included to document the complete planned search dictionary.

## Table S3. Strict Drug-Linked Tier 1 Confirmatory Analysis for BJI-Supported Signals

| **Signal (drug – ADE category)** | **Main analysis BJI ROR (95% CI) N = 9,072** | Confirmatory — strict drug-linked Tier 1 ROR (95% CI) N = 8,309 (763 excluded) | **Δ ROR (%)** | **Retained after exclusion** |
| --- | --- | --- | --- | --- |
| Linezolid – Hematologic | 3.65 (3.21–4.16) | 3.96 (3.46–4.53) | +8.4% | Yes |
| Vancomycin – Nephrotoxicity | 3.30 (2.95–3.70) | 3.66 (3.24–4.13) | +10.7% | Yes |
| Ceftaroline – Hematologic | 4.81 (3.42–6.77) | 4.63 (3.24–6.62) | −3.7% | Yes |
| Ertapenem – Neurotoxicity | 4.76 (3.50–6.47) | 4.84 (3.54–6.61) | +1.7% | Yes |

Note 1 — Definitions — The main analysis cohort (N = 9,072) was defined per the Methods (Tier 1 — target antibiotic with a Tier 1 BJI MedDRA preferred term; Tier 2 — target antibiotic with a generic infection indication AND another drug in the same case with an orthopedic procedure indication; manuscript-stated split 8,944 Tier 1 plus 128 Tier 2). The strict drug-linked Tier 1 confirmatory analysis reported here uses a stricter drug-linked Tier 1 definition that further excludes 763 cases whose target-antibiotic drug record could not be matched to a Tier 1 indication-drug pair via the indi_drug_seq linkage.

Note 2 — Result — All four BJI-supported signals remained statistically supported (ROR lower 95% CI > 1.0 and exposed-event count ≥ 3) after strict drug-linked Tier 1 restriction, with absolute ROR change between −3.7% and +10.7%. Across all 28 BJI-retained signals from Supplementary Table S5, 28 of 28 remained retained under the strict drug-linked Tier 1 definition, with mean |Δ ROR| of 7.1% and maximum |Δ ROR| of 24.1%. (The <= 10.7% bound cited in the main text refers to the four headline signals; the 24.1% maximum applies across all 28 BJI-retained signals.)

Note 3 — Interpretation — The confirmatory analysis shows that the four BJI-supported signals (vancomycin–nephrotoxicity, linezolid–hematologic toxicity, ceftaroline–hematologic toxicity, ertapenem–neurotoxicity) are robust to alternative operational definitions of the Tier 1/Tier 2 split. Headline signals were essentially unchanged when Tier 2 (contextual-indication) cases were excluded under the strict drug-linked Tier 1 definition.

Note 4 — Confirmatory analyses in the strict drug-linked Tier 1 cohort (N = 8,309) — The prioritized signals were re-estimated in the strict drug-linked Tier 1 cohort across the same sensitivity dimensions reported for the main cohort, with concordant results. Primary-suspect-only (PS-only) re-analysis (n = 6,885 PS cases) preserved all four signals: vancomycin–nephrotoxicity ROR 4.20 (95% CI 3.66–4.82), linezolid–hematologic toxicity 4.13 (3.56–4.80), ceftaroline–hematologic toxicity 4.78 (3.05–7.47), and ertapenem–neurotoxicity 7.57 (5.38–10.65). Active-comparator analyses among mutually exclusive exposed cases likewise remained directionally consistent: vancomycin vs daptomycin (nephrotoxicity) 4.25 (3.54–5.11), daptomycin vs vancomycin (myotoxicity) 22.93 (14.35–36.66), linezolid vs vancomycin (hematologic) 3.32 (2.81–3.93), ceftaroline vs ceftriaxone (hematologic) 6.08 (3.91–9.46), and ertapenem vs meropenem (neurotoxicity) 6.63 (3.10–14.18). Daptomycin myotoxicity remained prominent (295 of 1,904 daptomycin cases, 15.5%; within-cohort ROR 6.81, 95% CI 5.59–8.29). Thus every prioritized signal and key sensitivity analysis is reproduced in the drug-linked Tier 1 cohort.

## Table S4. ADE Category-to-PT Mapping

| ADE Category | MedDRA Preferred Term |
| --- | --- |
| Nephrotoxicity | ACUTE KIDNEY INJURY |
| Nephrotoxicity | RENAL FAILURE |
| Nephrotoxicity | RENAL FAILURE ACUTE |
| Nephrotoxicity | RENAL IMPAIRMENT |
| Nephrotoxicity | NEPHROTOXICITY |
| Nephrotoxicity | BLOOD CREATININE INCREASED |
| Nephrotoxicity | RENAL TUBULAR NECROSIS |
| Hepatotoxicity | HEPATOTOXICITY |
| Hepatotoxicity | DRUG-INDUCED LIVER INJURY |
| Hepatotoxicity | HEPATIC FAILURE |
| Hepatotoxicity | HEPATITIS |
| Hepatotoxicity | LIVER FUNCTION TEST ABNORMAL |
| Hepatotoxicity | ALANINE AMINOTRANSFERASE INCREASED |
| Hepatotoxicity | ASPARTATE AMINOTRANSFERASE INCREASED |
| Hepatotoxicity | JAUNDICE |
| Hepatotoxicity | HEPATIC ENZYME INCREASED |
| Hematologic | THROMBOCYTOPENIA |
| Hematologic | NEUTROPENIA |
| Hematologic | PANCYTOPENIA |
| Hematologic | AGRANULOCYTOSIS |
| Hematologic | ANAEMIA |
| Hematologic | LEUKOPENIA |
| Hematologic | FEBRILE NEUTROPENIA |
| Hematologic | BONE MARROW FAILURE |
| Hematologic | EOSINOPHILIA |
| C. difficile | CLOSTRIDIUM DIFFICILE COLITIS |
| C. difficile | CLOSTRIDIUM DIFFICILE INFECTION |
| C. difficile | PSEUDOMEMBRANOUS COLITIS |
| C. difficile | CLOSTRIDIOIDES DIFFICILE INFECTION |
| Neurotoxicity | PERIPHERAL NEUROPATHY |
| Neurotoxicity | NEUROPATHY PERIPHERAL |
| Neurotoxicity | SEIZURE |
| Neurotoxicity | CONVULSION |
| Neurotoxicity | OPTIC NEURITIS |
| Neurotoxicity | ENCEPHALOPATHY |
| Neurotoxicity | SEROTONIN SYNDROME |
| Tendon/Musculoskeletal | TENDON RUPTURE |
| Tendon/Musculoskeletal | TENDINITIS |
| Tendon/Musculoskeletal | ACHILLES TENDON RUPTURE |
| Tendon/Musculoskeletal | TENDON DISORDER |
| Tendon/Musculoskeletal | MYOPATHY (interpreted separately) |
| Tendon/Musculoskeletal | RHABDOMYOLYSIS (interpreted separately) |
| Cardiovascular | QT PROLONGATION |
| Cardiovascular | ELECTROCARDIOGRAM QT PROLONGED |
| Cardiovascular | TORSADE DE POINTES |
| Cardiovascular | CARDIAC ARREST |
| Skin/Allergy | STEVENS-JOHNSON SYNDROME |
| Skin/Allergy | TOXIC EPIDERMAL NECROLYSIS |
| Skin/Allergy | DRESS |
| Skin/Allergy | RED MAN SYNDROME |
| Skin/Allergy | ANAPHYLACTIC REACTION |
| Skin/Allergy | RASH |
| Skin/Allergy | URTICARIA |
| Skin/Allergy | PRURITUS |
| Ototoxicity | DEAFNESS |
| Ototoxicity | HEARING IMPAIRED |
| Ototoxicity | TINNITUS |
| Ototoxicity | OTOTOXICITY |
| Ototoxicity | HYPOACUSIS |

Note — Tendon disorders and myotoxicity were treated as distinct constructs. Although rhabdomyolysis, myopathy, and related muscle terms appear under the broad musculoskeletal heading of the prespecified full-database preferred-term pool, they represent myotoxicity rather than tendon pathology and were therefore analysed and reported separately as a myotoxicity construct (Supplementary Tables S6 and S8); accordingly, the within-cohort Tendon/Musculoskeletal counts (Supplementary Table S5) do not include myotoxicity preferred terms, and the daptomycin musculoskeletal signal is reported as myotoxicity (within-BJI ROR 7.45). MedDRA version 27.0.

## Table S5. Within-BJI-Cohort Disproportionality Analysis

Within-cohort ROR computed using the 9,072 BJI cases as reference population. Classification — "BJI-retained" = within-cohort ROR lower 95% CI > 1 and a >= 3; "Directionally positive, not retained" = BJI-ROR > 1 but CI crosses 1 or a < 3; "Not supported in BJI" = BJI-ROR <= 1; "Sparse; estimate unstable" = a(BJI) = 0, continuity-corrected estimate not interpretable. Shown for all 103 full-FAERS robust signals. A daptomycin–myotoxicity construct is listed separately.

| **Drug** | **ADE** | **a(BJI)** | **BJI-ROR (95% CI)** | **FAERS-ROR** | **Classification** |
| --- | --- | --- | --- | --- | --- |
| Tedizolid | Tendon/MSK | 0 | Sparse* | 4.1 | Sparse; estimate unstable |
| Moxifloxacin | Tendon/MSK | 0 | Sparse* | 6.5 | Sparse; estimate unstable |
| Ceftriaxone | Tendon/MSK | 0 | Sparse* | 2.5 | Sparse; estimate unstable |
| Moxifloxacin | Cardiovascular | 6 | 12.41 (5.32-28.96) | 7.3 | BJI-retained |
| Ciprofloxacin | Tendon/MSK | 0 | Sparse* | 21.7 | Sparse; estimate unstable |
| Pip-Tazo | Tendon/MSK | 0 | Sparse* | 3.9 | Sparse; estimate unstable |
| Levofloxacin | Tendon/MSK | 0 | Sparse* | 29.1 | Sparse; estimate unstable |
| Rifampicin | Tendon/MSK | 0 | Sparse* | 3.8 | Sparse; estimate unstable |
| Linezolid | Tendon/MSK | 0 | Sparse* | 3.9 | Sparse; estimate unstable |
| Ceftaroline | Hematologic | 64 | 4.81 (3.42-6.77) | 13.3 | BJI-retained |
| Ertapenem | Neurotoxicity | 55 | 4.76 (3.50-6.47) | 10.6 | BJI-retained |
| Tedizolid | Hematologic | 26 | 4.63 (2.73-7.84) | 12.4 | BJI-retained |
| Moxifloxacin | Ototoxicity | 4 | 3.94 (1.50-10.33) | 6.4 | BJI-retained |
| Linezolid | Hematologic | 479 | 3.65 (3.21-4.16) | 12.5 | BJI-retained |
| Vancomycin | Skin/Allergy | 732 | 3.58 (3.18-4.03) | 5.6 | BJI-retained |
| Daptomycin | Tendon/MSK | 0 | Sparse* | 20.5 | Sparse; estimate unstable |
| Vancomycin | Nephrotoxicity | 785 | 3.30 (2.95-3.70) | 11.9 | BJI-retained |
| Meropenem | C. difficile | 12 | 3.29 (1.80-6.02) | 24.3 | BJI-retained |
| Linezolid | Neurotoxicity | 109 | 2.85 (2.25-3.60) | 6.9 | BJI-retained |
| Rifampicin | Hepatotoxicity | 70 | 2.70 (2.05-3.56) | 14.9 | BJI-retained |
| Ciprofloxacin | Ototoxicity | 18 | 2.60 (1.55-4.35) | 7.1 | BJI-retained |
| TMP-SMX | C. difficile | 12 | 2.50 (1.37-4.55) | 6.7 | BJI-retained |
| Cefazolin | C. difficile | 5 | 2.32 (0.97-5.53) | 25.6 | Directionally positive, not retained |
| Levofloxacin | C. difficile | 18 | 2.21 (1.32-3.68) | 7.7 | BJI-retained |
| TMP-SMX | Hepatotoxicity | 33 | 2.19 (1.51-3.18) | 10.3 | BJI-retained |
| Cefazolin | Cardiovascular | 2 | 2.12 (0.59-7.64) | 5.6 | Directionally positive, not retained |
| Ceftriaxone | Skin/Allergy | 145 | 2.04 (1.68-2.49) | 4.9 | BJI-retained |
| Rifampicin | Skin/Allergy | 235 | 1.98 (1.69-2.32) | 4.0 | BJI-retained |
| Pip-Tazo | Skin/Allergy | 197 | 1.86 (1.57-2.21) | 5.2 | BJI-retained |
| Cefazolin | Skin/Allergy | 55 | 1.82 (1.34-2.48) | 8.0 | BJI-retained |
| Cefiderocol | C. difficile | 0 | Sparse* | 5.4 | Sparse; estimate unstable |
| Tedizolid | Neurotoxicity | 3 | 1.71 (0.58-5.07) | 5.1 | Directionally positive, not retained |
| Rifampicin | Hematologic | 231 | 1.71 (1.46-2.00) | 5.4 | BJI-retained |
| TMP-SMX | Nephrotoxicity | 126 | 1.68 (1.36-2.07) | 5.5 | BJI-retained |
| Pip-Tazo | Hepatotoxicity | 42 | 1.66 (1.19-2.31) | 6.4 | BJI-retained |
| TMP-SMX | Skin/Allergy | 112 | 1.65 (1.33-2.05) | 7.3 | BJI-retained |
| Meropenem | Skin/Allergy | 87 | 1.65 (1.29-2.10) | 3.1 | BJI-retained |
| Ciprofloxacin | Neurotoxicity | 45 | 1.62 (1.17-2.23) | 6.0 | BJI-retained |
| Pip-Tazo | Nephrotoxicity | 197 | 1.59 (1.35-1.89) | 7.1 | BJI-retained |
| Pip-Tazo | C. difficile | 13 | 1.58 (0.89-2.82) | 26.3 | Directionally positive, not retained |
| Pip-Tazo | Hematologic | 191 | 1.58 (1.33-1.88) | 7.0 | BJI-retained |
| Vancomycin | Hepatotoxicity | 108 | 1.43 (1.12-1.81) | 3.6 | BJI-retained |
| Levofloxacin | Neurotoxicity | 41 | 1.31 (0.94-1.82) | 6.4 | Directionally positive, not retained |
| Ertapenem | C. difficile | 5 | 1.30 (0.55-3.10) | 25.9 | Directionally positive, not retained |
| Vancomycin | C. difficile | 33 | 1.29 (0.85-1.97) | 22.8 | Directionally positive, not retained |
| Ciprofloxacin | C. difficile | 10 | 1.24 (0.65-2.36) | 15.1 | Directionally positive, not retained |
| Levofloxacin | Cardiovascular | 5 | 1.21 (0.50-2.96) | 4.0 | Directionally positive, not retained |
| Meropenem | Cardiovascular | 2 | 1.21 (0.34-4.35) | 4.0 | Directionally positive, not retained |
| Dalbavancin | C. difficile | 2 | 1.21 (0.34-4.27) | 3.8 | Directionally positive, not retained |
| TMP-SMX | Hematologic | 96 | 1.20 (0.96-1.51) | 7.0 | Directionally positive, not retained |
| Ceftriaxone | Hepatotoxicity | 21 | 1.16 (0.74-1.81) | 6.1 | Directionally positive, not retained |
| Dalbavancin | Skin/Allergy | 32 | 1.14 (0.78-1.67) | 5.6 | Directionally positive, not retained |
| Pip-Tazo | Ototoxicity | 9 | 1.12 (0.57-2.20) | 2.3 | Directionally positive, not retained |
| Meropenem | Hematologic | 68 | 1.07 (0.82-1.40) | 6.9 | Directionally positive, not retained |
| Ciprofloxacin | Cardiovascular | 4 | 1.07 (0.40-2.82) | 2.6 | Directionally positive, not retained |
| Moxifloxacin | Skin/Allergy | 18 | 1.04 (0.63-1.71) | 4.1 | Directionally positive, not retained |
| Rifampicin | Nephrotoxicity | 166 | 1.03 (0.86-1.22) | 4.5 | Directionally positive, not retained |
| Ciprofloxacin | Nephrotoxicity | 137 | 1.02 (0.84-1.23) | 3.0 | Directionally positive, not retained |
| Moxifloxacin | Neurotoxicity | 4 | 1.00 (0.39-2.59) | 8.7 | Directionally positive, not retained |
| Cefazolin | Hepatotoxicity | 7 | 0.99 (0.47-2.06) | 4.3 | Not supported in BJI |
| Linezolid | Ototoxicity | 13 | 0.97 (0.54-1.74) | 3.3 | Not supported in BJI |
| Ceftriaxone | Hematologic | 91 | 0.97 (0.77-1.22) | 5.2 | Not supported in BJI |
| Vancomycin | Hematologic | 419 | 0.97 (0.85-1.09) | 4.5 | Not supported in BJI |
| Levofloxacin | Nephrotoxicity | 143 | 0.96 (0.80-1.16) | 2.2 | Not supported in BJI |
| Ertapenem | Hepatotoxicity | 12 | 0.95 (0.53-1.69) | 2.9 | Not supported in BJI |
| Levofloxacin | Skin/Allergy | 123 | 0.92 (0.75-1.12) | 2.7 | Not supported in BJI |
| Linezolid | Cardiovascular | 6 | 0.90 (0.39-2.05) | 7.5 | Not supported in BJI |
| Ceftriaxone | C. difficile | 5 | 0.89 (0.37-2.11) | 19.4 | Not supported in BJI |
| Moxifloxacin | Hepatotoxicity | 3 | 0.88 (0.30-2.58) | 8.3 | Not supported in BJI |
| Meropenem | Hepatotoxicity | 11 | 0.87 (0.48-1.59) | 5.3 | Not supported in BJI |
| Tedizolid | Ototoxicity | 0 | Sparse* | 5.5 | Sparse; estimate unstable |
| Ciprofloxacin | Skin/Allergy | 106 | 0.85 (0.69-1.05) | 3.4 | Not supported in BJI |
| Daptomycin | Hepatotoxicity | 59 | 0.84 (0.63-1.12) | 3.9 | Not supported in BJI |
| Ceftriaxone | Nephrotoxicity | 83 | 0.84 (0.66-1.06) | 3.9 | Not supported in BJI |
| Ceftriaxone | Cardiovascular | 2 | 0.82 (0.23-2.94) | 4.7 | Not supported in BJI |
| Cefazolin | Nephrotoxicity | 32 | 0.80 (0.55-1.17) | 2.6 | Not supported in BJI |
| Ceftaroline | Hepatotoxicity | 3 | 0.78 (0.27-2.26) | 3.5 | Not supported in BJI |
| Pip-Tazo | Cardiovascular | 3 | 0.77 (0.26-2.30) | 3.7 | Not supported in BJI |
| Linezolid | C. difficile | 11 | 0.76 (0.41-1.41) | 7.0 | Not supported in BJI |
| Ceftaroline | Skin/Allergy | 15 | 0.73 (0.43-1.24) | 3.6 | Not supported in BJI |
| Ciprofloxacin | Hematologic | 101 | 0.72 (0.58-0.89) | 2.4 | Not supported in BJI |
| Ceftaroline | Nephrotoxicity | 16 | 0.68 (0.41-1.14) | 7.3 | Not supported in BJI |
| Ciprofloxacin | Hepatotoxicity | 18 | 0.67 (0.42-1.08) | 2.7 | Not supported in BJI |
| Cefazolin | Hematologic | 26 | 0.66 (0.44-0.99) | 2.8 | Not supported in BJI |
| TMP-SMX | Neurotoxicity | 12 | 0.63 (0.36-1.11) | 2.3 | Not supported in BJI |
| Levofloxacin | Hematologic | 99 | 0.63 (0.50-0.78) | 2.4 | Not supported in BJI |
| Meropenem | Nephrotoxicity | 44 | 0.61 (0.45-0.84) | 4.3 | Not supported in BJI |
| Moxifloxacin | Hematologic | 12 | 0.59 (0.33-1.07) | 2.8 | Not supported in BJI |
| Linezolid | Hepatotoxicity | 28 | 0.59 (0.40-0.88) | 5.9 | Not supported in BJI |
| Levofloxacin | Ototoxicity | 5 | 0.59 (0.25-1.39) | 3.1 | Not supported in BJI |
| Daptomycin | Hematologic | 230 | 0.58 (0.50-0.67) | 6.5 | Not supported in BJI |
| Vancomycin | Cardiovascular | 8 | 0.53 (0.25-1.12) | 2.5 | Not supported in BJI |
| Daptomycin | Skin/Allergy | 195 | 0.52 (0.45-0.61) | 2.3 | Not supported in BJI |
| Linezolid | Nephrotoxicity | 139 | 0.52 (0.44-0.63) | 3.8 | Not supported in BJI |
| Daptomycin | Nephrotoxicity | 217 | 0.51 (0.44-0.60) | 5.0 | Not supported in BJI |
| Cefiderocol | Nephrotoxicity | 2 | 0.50 (0.14-1.84) | 4.6 | Not supported in BJI |
| Daptomycin | C. difficile | 10 | 0.41 (0.21-0.77) | 4.0 | Not supported in BJI |
| Rifampicin | C. difficile | 4 | 0.40 (0.16-1.03) | 4.2 | Not supported in BJI |
| Moxifloxacin | C. difficile | 0 | Sparse* | 3.7 | Sparse; estimate unstable |
| Levofloxacin | Hepatotoxicity | 11 | 0.37 (0.20-0.66) | 3.0 | Not supported in BJI |
| Dalbavancin | Nephrotoxicity | 13 | 0.36 (0.21-0.63) | 3.2 | Not supported in BJI |
| Ertapenem | Hematologic | 25 | 0.34 (0.22-0.51) | 4.3 | Not supported in BJI |
| Tedizolid | Hepatotoxicity | 0 | Sparse* | 3.0 | Sparse; estimate unstable |
| Daptomycin | Myotoxicity | 330 | 7.45 (6.16-9.02) | — | BJI-retained (separate myotoxicity construct) |

* When a(BJI) = 0, the continuity-corrected ROR estimate is mathematically unstable and should not be interpreted as evidence of a signal. These entries are retained but classified as "Sparse; estimate unstable."

## Table S6. Daptomycin Myotoxicity-Related Preferred-Term Distribution — Full-FAERS vs BJI Cohort

| **MedDRA Preferred Term** | **PT pool category** | **Full-FAERS Daptomycin n unique cases** | **BJI cohort Daptomycin n unique cases** |
| --- | --- | --- | --- |
| BLOOD CREATINE PHOSPHOKINASE INCREASED | Extended myotoxicity | 560 | 173 |
| RHABDOMYOLYSIS | Original Tendon/MSK pool* | 466 | 121 |
| MYALGIA | Extended myotoxicity | 146 | 47 |
| MYOSITIS | Extended myotoxicity | 61 | 20 |
| MUSCULAR WEAKNESS | Extended myotoxicity | 52 | 11 |
| MUSCLE SPASMS | Extended myotoxicity | 31 | 10 |
| MYOPATHY | Original Tendon/MSK pool | 31 | 12 |
| MUSCLE DISORDER | Extended myotoxicity | 12 | 1 |
| MUSCLE TWITCHING | Extended myotoxicity | 11 | 5 |
| MUSCULOSKELETAL PAIN | Extended myotoxicity | 11 | 2 |
| TENDON RUPTURE | Original Tendon/MSK pool | 4 | 1 |
| MUSCULOSKELETAL DISCOMFORT | Extended myotoxicity | 3 | 0 |
| TENDON DISORDER | Original Tendon/MSK pool | 2 | 0 |
| MUSCLE TIGHTNESS | Extended myotoxicity | 1 | 1 |
| ACHILLES TENDON RUPTURE | Original Tendon/MSK pool | 0 | 0 |
| TENDINITIS | Original Tendon/MSK pool | 0 | 0 |
| *Total unique cases (any PT in original pool, 6 PTs)* | *—* | *499* | *132* |
| *Total unique cases (any PT in extended pool, 12 PTs)* | *—* | *756* | *225* |
| *Total unique cases (any PT in either pool)* | *—* | *1,158* | *330* |
| *Daptomycin total cases (denominator)* | *—* | *12,694* | *2,052* |

* RHABDOMYOLYSIS is listed in the prespecified Tendon/MSK preferred-term pool (Supplementary Table S4) but, per the Methods, was operationally interpreted separately as a distinct myotoxic reaction. The within-cohort disproportionality count in Supplementary Table S5 for Daptomycin–Tendon/MSK (a = 0) reflects this operational restriction — RHABDOMYOLYSIS, MYOPATHY, and other myotoxicity preferred terms were not additively counted in the within-cohort Tendon/MSK exposed-event count, even though they appear in the prespecified category definition. Table S6 above provides the unrestricted preferred-term breakdown.

Note — Interpretation — In the BJI cohort, daptomycin-treated cases reported myotoxicity-related preferred terms in 330 unique cases (16.1% of the 2,052 daptomycin BJI cases) when both the original Tendon/MSK pool and extended myotoxicity preferred terms are considered. The most frequent terms were BLOOD CREATINE PHOSPHOKINASE INCREASED (n = 173) and RHABDOMYOLYSIS (n = 121), consistent with the well-described creatine-kinase-elevation/rhabdomyolysis spectrum of daptomycin myotoxicity. The proportion of daptomycin cases with any myotoxicity preferred term in the BJI cohort (16.1%) is higher than in the full-FAERS daptomycin population (9.1%; 1,158 of 12,694), consistent with the longer mean therapy course typical of BJI management and reinforcing routine creatine-kinase monitoring during prolonged daptomycin therapy as recommended by current product labelling and clinical guidance.

**Table S7. Drug-Role Attribution and PS-Only Sensitivity for Prioritized Signals**

PS-only estimates test whether prioritized signals persist when antibiotic exposure is restricted to primary-suspect reports.

| **Drug** | **ADE category** | **Main PS/SS a** | **Main PS/SS ROR (95% CI)** | **PS-only a** | **PS-only ROR (95% CI)** | **PS share among exposed ADE cases** | **Primary interpretation** |
| --- | --- | --- | --- | --- | --- | --- | --- |
| Vancomycin | Nephrotoxicity | 785 | 3.30 (2.95-3.70) | 546 | 3.85 (3.38-4.40) | 546/785 (69.6%) | PS-supported |
| Linezolid | Hematologic | 479 | 3.65 (3.21-4.16) | 386 | 3.87 (3.35-4.47) | 386/479 (80.6%) | PS-supported |
| Ceftaroline | Hematologic | 64 | 4.81 (3.42-6.77) | 42 | 5.24 (3.42-8.01) | 42/64 (65.6%) | PS-supported |
| Ertapenem | Neurotoxicity | 55 | 4.76 (3.50-6.47) | 50 | 7.23 (5.18-10.10) | 50/55 (90.9%) | PS-supported |
| Vancomycin | Myotoxicity | 32 | 0.16 (0.11-0.23) | 19 | 0.13 (0.08-0.20) | 19/32 (59.4%) | Role-ambiguous / sparse under PS-only |
| Daptomycin | Myotoxicity | 330 | 7.45 (6.16-9.02) | 291 | 7.08 (5.80-8.63) | 291/330 (88.2%) | PS-supported |

**Table S8. Exploratory Active-Comparator Analysis within the BJI Cohort**

Mutually exclusive exposed cases were compared to provide indication-contextualized active-comparator evidence. Estimates remain exploratory because FAERS cannot fully adjust for severity, renal function, dose, duration, or concomitant therapy.

| **Index drug** | **Comparator** | **ADE category** | **Eligible mutually exclusive cases** | **Index exposed events** | **Comparator exposed events** | **Active-comparator ROR (95% CI)** | **Interpretation** |
| --- | --- | --- | --- | --- | --- | --- | --- |
| Vancomycin | Daptomycin | Nephrotoxicity | 4275 | 756 | 188 | 4.06 (3.42-4.83) | Higher reporting with index drug |
| Daptomycin | Vancomycin | Myotoxicity | 4275 | 318 | 20 | 24.64 (15.61-38.89) | Higher reporting with index drug |
| Linezolid | Vancomycin | Hematologic | 3731 | 431 | 371 | 3.05 (2.60-3.59) | Higher reporting with index drug |
| Ceftaroline | Ceftriaxone | Hematologic | 702 | 64 | 91 | 4.79 (3.19-7.20) | Higher reporting with index drug |
| Ertapenem | Meropenem | Neurotoxicity | 745 | 53 | 10 | 5.96 (2.98-11.91) | Higher reporting with index drug |

**Table S9. Infective-Endocarditis (IE) Long-Course Comparator Cohort**

IE comparator cohort (N = 1,500) constructed identically to the bacterial-pneumonia (BP) cohort from 26 endocarditis-spectrum MedDRA preferred terms (non-infective variants excluded; per-drug exposed cases: vancomycin 1,118, linezolid 273, ceftaroline 150, ertapenem 34), using the same continuity-corrected within-cohort ROR estimator and ADE preferred-term pools. Three of four prioritized signals remained elevated in IE as in BJI (both long-course infections), whereas they were attenuated in short-course pneumonia (Table 1, Figure 7); the ertapenem-IE estimate was sparse (exposed events a = 1) and is not interpretable.

| **Drug – ADE category** | **IE ROR (95% CI)** | **IE exposed a** | **IE drug cases** | **BJI ROR (95% CI)** | **BJI/IE ratio** |
| --- | --- | --- | --- | --- | --- |
| Linezolid – Hematologic | 2.75 (2.00-3.78) | 74 | 273 | 3.65 (3.21-4.16) | 1.33 |
| Vancomycin – Nephrotoxicity | 2.71 (1.84-3.98) | 228 | 1118 | 3.30 (2.95-3.70) | 1.22 |
| Ceftaroline – Hematologic | 2.66 (1.81-3.92) | 43 | 150 | 4.81 (3.42-6.77) | 1.81 |
| Ertapenem – Neurotoxicity | 1.56 (0.21-11.78) | 1 | 34 | 4.76 (3.50-6.47) | — |

## Table S10. Complete List of 38 Features Used in the Exploratory XGBoost Model

| **#** | **Feature name** | **Type** | **Feature category** | **Coding / units** | **Missing (%)** | **Handling of missingness** |
| --- | --- | --- | --- | --- | --- | --- |
| 1 | Vancomycin | Binary | Drug exposure | 1 = case included vancomycin (PS/SS); 0 otherwise | 0 | — |
| 2 | Daptomycin | Binary | Drug exposure | 1/0 | 0 | — |
| 3 | Linezolid | Binary | Drug exposure | 1/0 | 0 | — |
| 4 | Tedizolid | Binary | Drug exposure | 1/0 | 0 | — |
| 5 | Rifampicin | Binary | Drug exposure | 1/0 | 0 | — |
| 6 | Ciprofloxacin | Binary | Drug exposure | 1/0 | 0 | — |
| 7 | Levofloxacin | Binary | Drug exposure | 1/0 | 0 | — |
| 8 | Moxifloxacin | Binary | Drug exposure | 1/0 | 0 | — |
| 9 | TMP-SMX | Binary | Drug exposure | 1/0 | 0 | — |
| 10 | Ceftriaxone | Binary | Drug exposure | 1/0 | 0 | — |
| 11 | Cefazolin | Binary | Drug exposure | 1/0 | 0 | — |
| 12 | Ceftaroline | Binary | Drug exposure | 1/0 | 0 | — |
| 13 | Piperacillin–tazobactam | Binary | Drug exposure | 1/0 | 0 | — |
| 14 | Meropenem | Binary | Drug exposure | 1/0 | 0 | — |
| 15 | Ertapenem | Binary | Drug exposure | 1/0 | 0 | — |
| 16 | Dalbavancin | Binary | Drug exposure | 1/0 | 0 | — |
| 17 | Cefiderocol | Binary | Drug exposure | 1/0 | 0 | — |
| 18 | Age (years) | Continuous | Biologic | Years | 18.2 | Median imputation (65 y) + missingness indicator |
| 19 | Age squared | Derived | Biologic | Years² | — | Computed from imputed age |
| 20 | Sex (male) | Binary | Biologic | 1 = male; 0 = female/unknown | 0 | — |
| 21 | Sex (unknown) | Binary | Biologic | 1 = unknown/missing sex; 0 otherwise | 0 | — |
| 22 | Body weight (kg) | Continuous | Biologic | Kilograms (converted from lb where applicable) | 62.4 | Median imputation + missingness indicator |
| 23 | Intravenous route | Binary | Drug exposure | 1 = any drug record coded IV | 0 | — |
| 24 | Oral route | Binary | Drug exposure | 1 = any drug record coded oral | 0 | — |
| 25 | Total drugs per case | Count | Drug exposure | Integer | 0 | — |
| 26 | Total target ABX count | Count | Drug exposure | Number of target antibiotics in the case (0–17) | 0 | — |
| 27 | Number of reactions per case | Count | Reporting metadata | Integer | 0 | — |
| 28 | Therapy duration (days) | Continuous | Drug exposure | Days; max across drug-therapy episodes | 54.1 | Median imputation + missingness indicator |
| 29 | Report year | Continuous | Reporting metadata | Calendar year | 0 | — |
| 30 | US report | Binary | Reporting metadata | 1 = reporter_country = US | 0 | — |
| 31 | Physician reporter | Binary | Reporting metadata | 1 = occp_cod ∈ {MD, PH, other HCP} | 0 | — |
| 32 | Consumer reporter | Binary | Reporting metadata | 1 = occp_cod = CN | 0 | — |
| 33 | Lawyer reporter | Binary | Reporting metadata | 1 = occp_cod = LW | 0 | — |
| 34 | Initial report (i_f_code = I) | Binary | Reporting metadata | 1/0 | 0 | — |
| 35 | Has PS role for a target ABX | Binary | Reporting metadata | 1 = at least one target antibiotic has PS role | 0 | — |
| 36 | Sepsis indication present | Binary | Drug exposure | 1 = sepsis-related PT in INDI table | 0 | — |
| 37 | Comorbid infection indication | Binary | Drug exposure | 1 = ≥1 additional infection indication beyond BJI | 0 | — |
| 38 | Treatment complexity (concomitant × duration) | Derived | Drug exposure | Total concomitant drugs × log1p(duration) | — | — |

Note — Feature numbering corresponds to the order shown in Figure 5 Panel A. Missingness rates are computed within the 9,072 BJI cohort cases. Binary indicators have no intrinsic missingness because absence implies 0.

## Table S11. Missingness Rates and Handling for Key Modelling Variables

| **Variable** | **n missing** | **% missing** | **Handling strategy** |
| --- | --- | --- | --- |
| Age (age_years) | 1,655 | 18.2% | Median imputation (cohort median 65 years); paired missingness indicator (binary) |
| Sex | 1,299 | 14.3% | Encoded as separate male / unknown indicators; no imputation |
| Body weight (kg) | 5,661 | 62.4% | Median imputation (cohort median 75 kg); paired missingness indicator |
| Therapy duration (days) | 4,907 | 54.1% | Median imputation across drug-therapy episodes (median 14 days); paired missingness indicator |
| Event date (event_dt) | 3,925 | 43.3% | Used only in TTO subset (n = 4,168 cases with complete therapy + event dates) |
| Therapy start (start_dt) | 3,779 | 41.7% | Same as above |
| Reporter country | 412 | 4.5% | Recoded as US/non-US/unknown three-level indicator |
| Reporter occupation | 1,083 | 11.9% | Recoded as physician/consumer/lawyer/other/unknown indicators |
| Route | 3,851 | 22.5% of drug records | Recoded into IV/oral/other binary indicators at case level |

Note — Missingness percentages for case-level variables use 9,072 as the denominator; for drug-level variables (route) the denominator is the 17,092 cohort drug records. TTO analyses were restricted to records with non-missing event_dt and start_dt and were therefore not imputed.

## Table S12. XGBoost Hyperparameters and Cross-Validation Configuration

| **Parameter** | **Value** | **Rationale / source** |
| --- | --- | --- |
| Objective | binary logistic | Binary outcome (severe vs non-severe) |
| Evaluation metric | auc, aucpr | Reported as ROC-AUC and PR-AUC |
| max_depth | 6 | Default-equivalent depth; selected via 3-fold inner grid search over {4, 6, 8} |
| learning_rate (eta) | 0.05 | Conservative shrinkage for stable feature importance ranking |
| n_estimators | 500 | With early stopping (rounds = 50) on inner validation fold |
| subsample | 0.8 | Row subsampling to reduce overfitting |
| colsample_bytree | 0.8 | Column subsampling per tree |
| min_child_weight | 1 | Default |
| gamma | 0 | Default |
| reg_alpha | 0 | Default |
| reg_lambda | 1 | Default L2 regularization |
| scale_pos_weight | 5.48 | Negative-to-positive class ratio (7,671 / 1,401) |
| tree_method | hist | Memory-efficient histogram method |
| random_state | 42 | Reproducibility seed |
| Cross-validation | Stratified 5-fold | Stratified by outcome label |
| Inner hyperparameter selection | 3-fold grid on training folds only | Avoids information leakage into reported metrics |
| SHAP backend | TreeExplainer | Model-specific exact SHAP values |
| Feature standardization | Not applied | Tree-based model invariant to monotone transformations |

Note — All hyperparameters were prespecified before the final outer-loop cross-validation. The reported performance metrics (ROC-AUC, PR-AUC, sensitivity, specificity, PPV) are aggregated across the five outer test folds. The model was used only for exploratory association analysis (feature ranking) and was not calibrated for individual-level clinical prediction.

## Table S13. Baseline Comparison of TTO-Evaluable Cases versus Missing-Date Subset

| **Characteristic** | **TTO-evaluable (n = 4,761)** | **Missing-date subset (n = 4,311)** | **Comment** |
| --- | --- | --- | --- |
| Age, median years | 67 | 64 | TTO-evaluable modestly older |
| Male sex | 2,560 (53.8%) | 2,136 (49.5%) | Slightly more male in TTO-evaluable |
| Female sex | 1,711 (35.9%) | 1,366 (31.7%) | Slightly more female in TTO-evaluable |
| Unknown sex | 490 (10.3%) | 809 (18.8%) | Missing-date subset has higher missing-sex (consistent with documentation incompleteness) |
| Serious outcome (DE/LT/HO) | 3,077 (64.6%) | 2,226 (51.6%) | TTO-evaluable enriched for serious cases |
| Physician reporter | 1,573 (33.0%) | 1,282 (29.7%) | Modestly more physician-reported |
| Consumer reporter | 374 (7.9%) | 349 (8.1%) | Modestly more consumer-reported |
| Vancomycin (most-reported) | 938 (19.7%) | 1,182 (27.4%) | Missing-date subset disproportionately vancomycin |
| Daptomycin (2nd-most) | 937 (19.7%) | 854 (19.8%) | Comparable across subsets |
| Linezolid (3rd-most) | 635 (13.3%) | 550 (12.8%) | Comparable across subsets |

Note 1 — Cohort split — TTO-evaluable = BJI cases (n = 4,761) with both a valid (parseable) DEMO.event_dt and at least one valid (parseable) THER.start_dt. Missing-date subset = BJI cases (n = 4,311) failing one or both of these criteria. The published TTO analytic subset (n = 4,168) is a strict subset of the 4,761 TTO-evaluable cases after applying the prespecified 0 < TTO ≤ 3,650 days filter.

Note 2 — Interpretation — The TTO-evaluable subset is enriched for older patients, serious outcomes, and physician-reported cases relative to the missing-date subset. This is consistent with documentation-completeness selection (more thoroughly documented serious cases retain valid timing fields) rather than systematic clinical divergence in disease severity or antibiotic class. The Weibull TTO findings should therefore be interpreted as descriptive reporting patterns within a documentation-enriched subset, not as incidence-based timing estimates generalisable to the full BJI cohort or to the broader population of antibiotic-treated BJI patients.

Note (applies to Supplementary Tables S1, S5, S15, and S16 and main Table 3) — Pip-Tazo = piperacillin–tazobactam; TMP-SMX = trimethoprim–sulfamethoxazole.

**Table S14. TTO Sensitivity Analysis Using Earliest Valid Onset Definitions**

This conservative sensitivity analysis uses the subset of TTO records that could be strictly linked through primaryid and drug_seq, then evaluates whether earliest valid onset definitions materially influence descriptive Weibull interpretation. These estimates should be interpreted cautiously because date-complete reports are documentation-enriched and this strict linkage subset is smaller than the broader TTO set reported in the main analysis.

| **Analysis set** | **Drug** | **n** | **Median TTO (IQR), days** | **Shape α (95% CI)** | **Scale β** | **Failure type** |
| --- | --- | --- | --- | --- | --- | --- |
| Strict drug-sequence-linked TTO episodes | Overall | 933 | 14 (6-28) | 0.774 (0.732-0.839) | 25.1 | Early failure |
| Earliest valid TTO per case | Overall | 749 | 14 (6-26) | 0.789 (0.727-0.874) | 23.3 | Early failure |
| Earliest valid TTO per drug-case | Vancomycin | 180 | 14 (7-24) | 1.211 (1.075-1.450) | 19.7 | Wear-out |
| Earliest valid TTO per drug-case | Daptomycin | 176 | 12 (7-22) | 0.904 (0.805-1.109) | 19.7 | Indeterminate |
| Earliest valid TTO per drug-case | Linezolid | 150 | 17 (6-40) | 0.680 (0.608-0.788) | 36.6 | Early failure |
| Earliest valid TTO per drug-case | Ertapenem | 41 | 9 (4-19) | 1.134 (0.999-1.389) | 14.1 | Indeterminate |

## Table S15. PS-Only Sensitivity Analysis (Top 20 Signals)

| Drug | ADE | a(PS) | PS-Only ROR (95% CI) | Full-FAERS ROR |
| --- | --- | --- | --- | --- |
| Levofloxacin | Tendon/MSK | 0 | 13.36 (0.26-674.21) | 29.11 |
| Pip-Tazo | C. difficile | 3 | 1.54 (0.52-4.57) | 26.26 |
| Ertapenem | C. difficile | 4 | 2.48 (0.94-6.57) | 25.90 |
| Cefazolin | C. difficile | 2 | 4.20 (1.16-15.22) | 25.63 |
| Meropenem | C. difficile | 2 | 3.21 (0.89-11.58) | 24.28 |
| Vancomycin | C. difficile | 11 | 0.89 (0.46-1.70) | 22.78 |
| Ciprofloxacin | Tendon/MSK | 0 | 14.04 (0.28-708.16) | 21.70 |
| Daptomycin | Tendon/MSK | 0 | 3.52 (0.07-177.54) | 20.54 |
| Ceftriaxone | C. difficile | 1 | 0.89 (0.18-4.56) | 19.43 |
| Ciprofloxacin | C. difficile | 7 | 2.33 (1.07-5.08) | 15.10 |
| Rifampicin | Hepatotoxicity | 24 | 2.41 (1.57-3.72) | 14.89 |
| Ceftaroline | Hematologic | 42 | 5.24 (3.42-8.01) | 13.34 |
| Linezolid | Hematologic | 386 | 3.87 (3.35-4.47) | 12.48 |
| Tedizolid | Hematologic | 15 | 5.75 (2.80-11.80) | 12.35 |
| Vancomycin | Nephrotoxicity | 546 | 3.85 (3.38-4.40) | 11.86 |
| Ertapenem | Neurotoxicity | 50 | 7.23 (5.18-10.10) | 10.58 |
| TMP-SMX | Hepatotoxicity | 5 | 1.25 (0.53-2.95) | 10.33 |
| Moxifloxacin | Neurotoxicity | 2 | 0.84 (0.24-2.96) | 8.75 |
| Moxifloxacin | Hepatotoxicity | 3 | 1.37 (0.47-4.04) | 8.25 |
| Cefazolin | Skin/Allergy | 23 | 3.48 (2.16-5.62) | 8.00 |

Note for Table S15 — Where PS-only a = 0, continuity-corrected ROR estimates are unstable and should not be interpreted as evidence of a PS-only signal.

## Table S16. Complete Disproportionality Results (N=20,006,981 background)

| Drug | ADE | a | ROR (95% CI) | PRR | IC (95% CI) | EB05 | Robust |
| --- | --- | --- | --- | --- | --- | --- | --- |
| Cefazolin | C. difficile | 199 | 25.63 (22.25-29.54) | 24.74 | 4.62 (4.42-4.82) | 21.88 | Yes |
| Cefazolin | Cardiovascular | 142 | 5.63 (4.77-6.65) | 5.51 | 2.46 (2.22-2.70) | 4.80 | Yes |
| Cefazolin | Hematologic | 317 | 2.81 (2.51-3.14) | 2.70 | 1.43 (1.28-1.59) | 2.46 | Yes |
| Cefazolin | Hepatotoxicity | 252 | 4.31 (3.80-4.89) | 4.16 | 2.05 (1.88-2.23) | 3.74 | Yes |
| Cefazolin | Nephrotoxicity | 267 | 2.64 (2.34-2.99) | 2.56 | 1.36 (1.18-1.53) | 2.32 | Yes |
| Cefazolin | Neurotoxicity | 101 | 1.17 (0.96-1.43) | 1.17 | 0.23 (-0.05-0.51) | 0.99 | No |
| Cefazolin | Ototoxicity | 19 | 0.75 (0.48-1.17) | 0.75 | -0.42 (-1.06-0.22) | 0.52 | No |
| Cefazolin | Skin/Allergy | 1353 | 8.00 (7.52-8.51) | 6.28 | 2.65 (2.57-2.73) | 6.00 | Yes |
| Cefazolin | Tendon/MSK | 11 | 0.91 (0.51-1.62) | 0.91 | -0.14 (-0.97-0.70) | 0.56 | No |
| Cefiderocol | C. difficile | 5 | 5.41 (2.34-12.51) | 5.37 | 2.42 (1.22-3.63) | 2.66 | Yes |
| Cefiderocol | Cardiovascular | 7 | 2.31 (1.13-4.75) | 2.30 | 1.20 (0.17-2.23) | 1.26 | No |
| Cefiderocol | Hematologic | 30 | 2.11 (1.46-3.03) | 2.06 | 1.04 (0.53-1.55) | 1.53 | No |
| Cefiderocol | Hepatotoxicity | 18 | 2.45 (1.54-3.89) | 2.41 | 1.27 (0.61-1.93) | 1.64 | No |
| Cefiderocol | Nephrotoxicity | 56 | 4.58 (3.49-6.01) | 4.29 | 2.10 (1.72-2.48) | 3.44 | Yes |
| Cefiderocol | Neurotoxicity | 18 | 1.71 (1.08-2.71) | 1.69 | 0.76 (0.10-1.41) | 1.15 | No |
| Cefiderocol | Ototoxicity | 0 | 0.15 (0.01-2.42) | 0.15 | -2.72 (-6.72-1.28) | 0.01 | No |
| Cefiderocol | Skin/Allergy | 42 | 1.60 (1.17-2.18) | 1.56 | 0.64 (0.21-1.08) | 1.21 | No |
| Cefiderocol | Tendon/MSK | 3 | 2.20 (0.77-6.29) | 2.20 | 1.13 (-0.38-2.65) | 0.91 | No |
| Ceftaroline | C. difficile | 5 | 3.07 (1.33-7.10) | 3.06 | 1.61 (0.41-2.82) | 1.52 | No |
| Ceftaroline | Cardiovascular | 12 | 2.19 (1.26-3.83) | 2.18 | 1.13 (0.33-1.93) | 1.37 | No |
| Ceftaroline | Hematologic | 274 | 13.34 (11.66-15.26) | 10.56 | 3.40 (3.23-3.57) | 9.56 | Yes |
| Ceftaroline | Hepatotoxicity | 45 | 3.47 (2.58-4.67) | 3.38 | 1.76 (1.34-2.18) | 2.65 | Yes |
| Ceftaroline | Nephrotoxicity | 150 | 7.29 (6.14-8.64) | 6.51 | 2.70 (2.47-2.93) | 5.69 | Yes |
| Ceftaroline | Neurotoxicity | 25 | 1.34 (0.90-1.98) | 1.33 | 0.41 (-0.15-0.97) | 0.96 | No |
| Ceftaroline | Ototoxicity | 7 | 1.30 (0.64-2.67) | 1.30 | 0.38 (-0.65-1.41) | 0.71 | No |
| Ceftaroline | Skin/Allergy | 155 | 3.58 (3.03-4.24) | 3.25 | 1.70 (1.48-1.93) | 2.85 | Yes |
| Ceftaroline | Tendon/MSK | 5 | 1.97 (0.85-4.56) | 1.97 | 0.98 (-0.23-2.18) | 0.98 | No |
| Ceftriaxone | C. difficile | 614 | 19.43 (17.92-21.07) | 18.92 | 4.21 (4.10-4.33) | 17.36 | Yes |
| Ceftriaxone | Cardiovascular | 490 | 4.75 (4.34-5.19) | 4.66 | 2.22 (2.09-2.34) | 4.31 | Yes |
| Ceftriaxone | Hematologic | 2292 | 5.23 (5.01-5.46) | 4.80 | 2.26 (2.20-2.32) | 4.62 | Yes |
| Ceftriaxone | Hepatotoxicity | 1421 | 6.08 (5.76-6.41) | 5.76 | 2.52 (2.44-2.59) | 5.48 | Yes |
| Ceftriaxone | Nephrotoxicity | 1562 | 3.88 (3.68-4.08) | 3.68 | 1.87 (1.80-1.95) | 3.52 | Yes |
| Ceftriaxone | Neurotoxicity | 483 | 1.37 (1.26-1.50) | 1.37 | 0.45 (0.32-0.58) | 1.27 | No |
| Ceftriaxone | Ototoxicity | 88 | 0.83 (0.67-1.02) | 0.83 | -0.26 (-0.57-0.04) | 0.70 | No |
| Ceftriaxone | Skin/Allergy | 3757 | 4.94 (4.77-5.11) | 4.28 | 2.09 (2.05-2.14) | 4.15 | Yes |
| Ceftriaxone | Tendon/MSK | 130 | 2.54 (2.14-3.02) | 2.54 | 1.34 (1.09-1.59) | 2.19 | Yes |
| Ciprofloxacin | C. difficile | 996 | 15.10 (14.17-16.10) | 14.81 | 3.84 (3.75-3.93) | 13.61 | Yes |
| Ciprofloxacin | Cardiovascular | 565 | 2.58 (2.37-2.80) | 2.56 | 1.35 (1.23-1.47) | 2.38 | Yes |
| Ciprofloxacin | Hematologic | 2354 | 2.42 (2.32-2.52) | 2.35 | 1.23 (1.17-1.28) | 2.26 | Yes |
| Ciprofloxacin | Hepatotoxicity | 1375 | 2.70 (2.56-2.85) | 2.65 | 1.40 (1.32-1.48) | 2.53 | Yes |
| Ciprofloxacin | Nephrotoxicity | 2550 | 2.97 (2.85-3.09) | 2.86 | 1.51 (1.46-1.57) | 2.76 | Yes |
| Ciprofloxacin | Neurotoxicity | 4110 | 6.03 (5.84-6.23) | 5.59 | 2.47 (2.42-2.51) | 5.39 | Yes |
| Ciprofloxacin | Ototoxicity | 1521 | 7.11 (6.76-7.49) | 6.92 | 2.77 (2.70-2.84) | 6.54 | Yes |
| Ciprofloxacin | Skin/Allergy | 5647 | 3.35 (3.26-3.45) | 3.07 | 1.61 (1.57-1.65) | 2.99 | Yes |
| Ciprofloxacin | Tendon/MSK | 2145 | 21.70 (20.76-22.69) | 20.76 | 4.31 (4.25-4.37) | 19.14 | Yes |
| Dalbavancin | C. difficile | 6 | 3.85 (1.78-8.32) | 3.83 | 1.94 (0.83-3.05) | 2.01 | Yes |
| Dalbavancin | Cardiovascular | 9 | 1.76 (0.93-3.34) | 1.76 | 0.81 (-0.10-1.73) | 1.03 | No |
| Dalbavancin | Hematologic | 50 | 2.10 (1.59-2.79) | 2.06 | 1.04 (0.64-1.44) | 1.63 | No |
| Dalbavancin | Hepatotoxicity | 15 | 1.22 (0.74-2.02) | 1.22 | 0.29 (-0.43-1.00) | 0.80 | No |
| Dalbavancin | Nephrotoxicity | 66 | 3.17 (2.48-4.06) | 3.05 | 1.61 (1.26-1.95) | 2.49 | Yes |
| Dalbavancin | Neurotoxicity | 15 | 0.85 (0.52-1.41) | 0.85 | -0.23 (-0.94-0.49) | 0.56 | No |
| Dalbavancin | Ototoxicity | 7 | 1.38 (0.67-2.83) | 1.38 | 0.46 (-0.57-1.49) | 0.76 | No |
| Dalbavancin | Skin/Allergy | 213 | 5.58 (4.81-6.48) | 4.73 | 2.24 (2.05-2.44) | 4.23 | Yes |
| Dalbavancin | Tendon/MSK | 1 | 0.57 (0.11-2.82) | 0.57 | -0.82 (-3.12-1.49) | 0.15 | No |
| Daptomycin | C. difficile | 60 | 4.00 (3.10-5.15) | 3.98 | 1.99 (1.63-2.35) | 3.22 | Yes |
| Daptomycin | Cardiovascular | 45 | 0.94 (0.70-1.25) | 0.94 | -0.09 (-0.51-0.33) | 0.73 | No |
| Daptomycin | Hematologic | 1273 | 6.47 (6.10-6.86) | 5.79 | 2.53 (2.45-2.61) | 5.52 | Yes |
| Daptomycin | Hepatotoxicity | 427 | 3.87 (3.51-4.27) | 3.75 | 1.91 (1.77-2.04) | 3.46 | Yes |
| Daptomycin | Nephrotoxicity | 911 | 5.02 (4.69-5.37) | 4.66 | 2.22 (2.12-2.31) | 4.41 | Yes |
| Daptomycin | Neurotoxicity | 233 | 1.45 (1.27-1.65) | 1.44 | 0.52 (0.34-0.71) | 1.29 | No |
| Daptomycin | Ototoxicity | 22 | 0.46 (0.30-0.69) | 0.46 | -1.12 (-1.71--0.52) | 0.33 | No |
| Daptomycin | Skin/Allergy | 868 | 2.25 (2.10-2.41) | 2.15 | 1.10 (1.00-1.20) | 2.03 | Yes |
| Daptomycin | Tendon/MSK | 461 | 20.54 (18.70-22.56) | 19.67 | 4.28 (4.15-4.42) | 18.04 | Yes |
| Ertapenem | C. difficile | 164 | 25.90 (22.16-30.28) | 24.99 | 4.64 (4.41-4.86) | 21.86 | Yes |
| Ertapenem | Cardiovascular | 26 | 1.26 (0.86-1.84) | 1.26 | 0.33 (-0.22-0.88) | 0.91 | No |
| Ertapenem | Hematologic | 383 | 4.29 (3.86-4.76) | 4.00 | 2.00 (1.86-2.15) | 3.68 | Yes |
| Ertapenem | Hepatotoxicity | 141 | 2.91 (2.47-3.45) | 2.85 | 1.51 (1.27-1.75) | 2.48 | Yes |
| Ertapenem | Nephrotoxicity | 124 | 1.47 (1.23-1.76) | 1.46 | 0.55 (0.29-0.80) | 1.26 | No |
| Ertapenem | Neurotoxicity | 649 | 10.58 (9.74-11.50) | 9.20 | 3.20 (3.09-3.31) | 8.61 | Yes |
| Ertapenem | Ototoxicity | 16 | 0.78 (0.48-1.26) | 0.78 | -0.36 (-1.06-0.33) | 0.52 | No |
| Ertapenem | Skin/Allergy | 241 | 1.39 (1.22-1.59) | 1.37 | 0.46 (0.27-0.64) | 1.23 | No |
| Ertapenem | Tendon/MSK | 9 | 0.92 (0.49-1.74) | 0.92 | -0.12 (-1.03-0.80) | 0.54 | No |
| Levofloxacin | C. difficile | 475 | 7.73 (7.06-8.47) | 7.66 | 2.92 (2.79-3.05) | 7.00 | Yes |
| Levofloxacin | Cardiovascular | 792 | 4.03 (3.75-4.32) | 3.97 | 1.98 (1.88-2.08) | 3.72 | Yes |
| Levofloxacin | Hematologic | 2117 | 2.40 (2.29-2.51) | 2.33 | 1.22 (1.15-1.28) | 2.24 | Yes |
| Levofloxacin | Hepatotoxicity | 1394 | 3.03 (2.88-3.20) | 2.97 | 1.56 (1.49-1.64) | 2.83 | Yes |
| Levofloxacin | Nephrotoxicity | 1729 | 2.19 (2.08-2.29) | 2.14 | 1.09 (1.03-1.16) | 2.05 | Yes |
| Levofloxacin | Neurotoxicity | 3910 | 6.36 (6.15-6.57) | 5.87 | 2.54 (2.49-2.58) | 5.66 | Yes |
| Levofloxacin | Ototoxicity | 616 | 3.09 (2.86-3.35) | 3.06 | 1.61 (1.49-1.72) | 2.85 | Yes |
| Levofloxacin | Skin/Allergy | 4163 | 2.65 (2.57-2.74) | 2.49 | 1.31 (1.27-1.36) | 2.42 | Yes |
| Levofloxacin | Tendon/MSK | 2545 | 29.11 (27.93-30.33) | 27.43 | 4.70 (4.64-4.75) | 25.13 | Yes |
| Linezolid | C. difficile | 195 | 7.04 (6.11-8.11) | 6.98 | 2.79 (2.59-3.00) | 6.17 | Yes |
| Linezolid | Cardiovascular | 651 | 7.52 (6.95-8.13) | 7.30 | 2.86 (2.75-2.97) | 6.80 | Yes |
| Linezolid | Hematologic | 4060 | 12.48 (12.05-12.92) | 10.04 | 3.32 (3.27-3.36) | 9.70 | Yes |
| Linezolid | Hepatotoxicity | 1180 | 5.92 (5.58-6.28) | 5.62 | 2.48 (2.40-2.57) | 5.33 | Yes |
| Linezolid | Nephrotoxicity | 1304 | 3.80 (3.59-4.02) | 3.61 | 1.85 (1.77-1.93) | 3.44 | Yes |
| Linezolid | Neurotoxicity | 1885 | 6.87 (6.55-7.21) | 6.29 | 2.65 (2.58-2.71) | 6.03 | Yes |
| Linezolid | Ototoxicity | 295 | 3.31 (2.95-3.71) | 3.27 | 1.71 (1.54-1.87) | 2.97 | Yes |
| Linezolid | Skin/Allergy | 1080 | 1.47 (1.38-1.56) | 1.44 | 0.53 (0.44-0.61) | 1.37 | No |
| Linezolid | Tendon/MSK | 171 | 3.95 (3.40-4.59) | 3.92 | 1.97 (1.75-2.18) | 3.45 | Yes |
| Meropenem | C. difficile | 503 | 24.28 (22.20-26.55) | 23.48 | 4.53 (4.40-4.66) | 21.47 | Yes |
| Meropenem | Cardiovascular | 274 | 4.01 (3.56-4.52) | 3.96 | 1.98 (1.81-2.15) | 3.58 | Yes |
| Meropenem | Hematologic | 1924 | 6.89 (6.56-7.23) | 6.12 | 2.61 (2.54-2.67) | 5.87 | Yes |
| Meropenem | Hepatotoxicity | 828 | 5.33 (4.97-5.72) | 5.09 | 2.34 (2.24-2.44) | 4.79 | Yes |
| Meropenem | Nephrotoxicity | 1125 | 4.27 (4.02-4.54) | 4.02 | 2.00 (1.92-2.09) | 3.82 | Yes |
| Meropenem | Neurotoxicity | 478 | 2.09 (1.91-2.29) | 2.06 | 1.04 (0.91-1.17) | 1.91 | No |
| Meropenem | Ototoxicity | 73 | 1.05 (0.84-1.32) | 1.05 | 0.07 (-0.26-0.40) | 0.87 | No |
| Meropenem | Skin/Allergy | 1676 | 3.14 (2.98-3.30) | 2.90 | 1.53 (1.46-1.60) | 2.78 | Yes |
| Meropenem | Tendon/MSK | 79 | 2.35 (1.89-2.93) | 2.35 | 1.23 (0.91-1.55) | 1.95 | No |
| Moxifloxacin | C. difficile | 96 | 3.73 (3.05-4.55) | 3.71 | 1.89 (1.60-2.18) | 3.13 | Yes |
| Moxifloxacin | Cardiovascular | 584 | 7.28 (6.70-7.91) | 7.07 | 2.81 (2.70-2.93) | 6.57 | Yes |
| Moxifloxacin | Hematologic | 1013 | 2.79 (2.62-2.98) | 2.69 | 1.43 (1.34-1.51) | 2.55 | Yes |
| Moxifloxacin | Hepatotoxicity | 1483 | 8.25 (7.82-8.70) | 7.64 | 2.93 (2.85-3.00) | 7.28 | Yes |
| Moxifloxacin | Nephrotoxicity | 416 | 1.25 (1.13-1.38) | 1.24 | 0.31 (0.17-0.45) | 1.15 | No |
| Moxifloxacin | Neurotoxicity | 2159 | 8.75 (8.36-9.15) | 7.80 | 2.95 (2.89-3.02) | 7.48 | Yes |
| Moxifloxacin | Ototoxicity | 519 | 6.40 (5.86-6.98) | 6.24 | 2.63 (2.51-2.76) | 5.78 | Yes |
| Moxifloxacin | Skin/Allergy | 2509 | 4.06 (3.90-4.24) | 3.63 | 1.86 (1.80-1.91) | 3.50 | Yes |
| Moxifloxacin | Tendon/MSK | 259 | 6.51 (5.76-7.36) | 6.43 | 2.68 (2.50-2.85) | 5.78 | Yes |
| Pip-Tazo | C. difficile | 818 | 26.26 (24.47-28.19) | 25.34 | 4.62 (4.53-4.72) | 23.29 | Yes |
| Pip-Tazo | Cardiovascular | 389 | 3.74 (3.38-4.14) | 3.69 | 1.88 (1.74-2.02) | 3.39 | Yes |
| Pip-Tazo | Hematologic | 2982 | 7.05 (6.78-7.32) | 6.24 | 2.63 (2.58-2.69) | 6.02 | Yes |
| Pip-Tazo | Hepatotoxicity | 1503 | 6.45 (6.12-6.79) | 6.08 | 2.60 (2.52-2.67) | 5.80 | Yes |
| Pip-Tazo | Nephrotoxicity | 2717 | 7.14 (6.86-7.44) | 6.40 | 2.67 (2.62-2.72) | 6.17 | Yes |
| Pip-Tazo | Neurotoxicity | 608 | 1.74 (1.60-1.88) | 1.72 | 0.78 (0.66-0.89) | 1.61 | No |
| Pip-Tazo | Ototoxicity | 238 | 2.26 (1.99-2.56) | 2.24 | 1.16 (0.98-1.35) | 2.01 | Yes |
| Pip-Tazo | Skin/Allergy | 3937 | 5.22 (5.04-5.40) | 4.48 | 2.16 (2.11-2.20) | 4.35 | Yes |
| Pip-Tazo | Tendon/MSK | 200 | 3.92 (3.41-4.51) | 3.90 | 1.96 (1.76-2.16) | 3.46 | Yes |
| Rifampicin | C. difficile | 96 | 4.24 (3.47-5.18) | 4.22 | 2.07 (1.79-2.36) | 3.56 | Yes |
| Rifampicin | Cardiovascular | 111 | 1.53 (1.27-1.84) | 1.53 | 0.61 (0.34-0.88) | 1.30 | No |
| Rifampicin | Hematologic | 1641 | 5.42 (5.15-5.71) | 4.96 | 2.31 (2.24-2.38) | 4.75 | Yes |
| Rifampicin | Hepatotoxicity | 2201 | 14.89 (14.23-15.58) | 12.93 | 3.68 (3.62-3.74) | 12.37 | Yes |
| Rifampicin | Nephrotoxicity | 1242 | 4.50 (4.25-4.77) | 4.22 | 2.07 (1.99-2.15) | 4.02 | Yes |
| Rifampicin | Neurotoxicity | 508 | 2.11 (1.93-2.31) | 2.08 | 1.05 (0.93-1.18) | 1.93 | No |
| Rifampicin | Ototoxicity | 154 | 2.11 (1.80-2.47) | 2.10 | 1.07 (0.84-1.30) | 1.84 | No |
| Rifampicin | Skin/Allergy | 2176 | 3.99 (3.82-4.18) | 3.57 | 1.83 (1.77-1.90) | 3.44 | Yes |
| Rifampicin | Tendon/MSK | 133 | 3.77 (3.18-4.47) | 3.75 | 1.90 (1.66-2.15) | 3.24 | Yes |
| TMP-SMX | C. difficile | 448 | 6.69 (6.09-7.35) | 6.64 | 2.71 (2.58-2.85) | 6.06 | Yes |
| TMP-SMX | Cardiovascular | 433 | 2.00 (1.82-2.20) | 1.99 | 0.99 (0.85-1.13) | 1.84 | No |
| TMP-SMX | Hematologic | 6082 | 7.00 (6.81-7.19) | 6.21 | 2.62 (2.58-2.65) | 6.01 | Yes |
| TMP-SMX | Hepatotoxicity | 4719 | 10.33 (10.02-10.65) | 9.38 | 3.20 (3.16-3.24) | 8.98 | Yes |
| TMP-SMX | Nephrotoxicity | 4425 | 5.50 (5.33-5.67) | 5.07 | 2.33 (2.29-2.37) | 4.90 | Yes |
| TMP-SMX | Neurotoxicity | 1675 | 2.35 (2.23-2.46) | 2.30 | 1.20 (1.13-1.26) | 2.20 | Yes |
| TMP-SMX | Ototoxicity | 382 | 1.75 (1.58-1.94) | 1.74 | 0.80 (0.66-0.94) | 1.60 | No |
| TMP-SMX | Skin/Allergy | 10608 | 7.34 (7.18-7.50) | 5.89 | 2.54 (2.51-2.57) | 5.73 | Yes |
| TMP-SMX | Tendon/MSK | 187 | 1.77 (1.53-2.04) | 1.76 | 0.82 (0.61-1.02) | 1.56 | No |
| Tedizolid | C. difficile | 3 | 2.98 (1.04-8.52) | 2.97 | 1.57 (0.06-3.08) | 1.23 | No |
| Tedizolid | Cardiovascular | 3 | 0.93 (0.33-2.66) | 0.93 | -0.10 (-1.61-1.41) | 0.39 | No |
| Tedizolid | Hematologic | 169 | 12.35 (10.43-14.64) | 9.95 | 3.31 (3.10-3.53) | 8.76 | Yes |
| Tedizolid | Hepatotoxicity | 25 | 2.95 (1.99-4.38) | 2.89 | 1.53 (0.97-2.09) | 2.09 | Yes |
| Tedizolid | Nephrotoxicity | 37 | 2.55 (1.84-3.54) | 2.47 | 1.31 (0.85-1.77) | 1.89 | No |
| Tedizolid | Neurotoxicity | 60 | 5.12 (3.94-6.65) | 4.81 | 2.27 (1.90-2.63) | 3.89 | Yes |
| Tedizolid | Ototoxicity | 20 | 5.54 (3.57-8.59) | 5.42 | 2.44 (1.81-3.06) | 3.77 | Yes |
| Tedizolid | Skin/Allergy | 40 | 1.31 (0.95-1.80) | 1.29 | 0.37 (-0.07-0.82) | 1.00 | No |
| Tedizolid | Tendon/MSK | 7 | 4.12 (2.01-8.46) | 4.09 | 2.03 (1.00-3.07) | 2.24 | Yes |
| Vancomycin | C. difficile | 1137 | 22.78 (21.45-24.20) | 22.10 | 4.41 (4.33-4.50) | 20.27 | Yes |
| Vancomycin | Cardiovascular | 425 | 2.52 (2.29-2.78) | 2.50 | 1.32 (1.18-1.46) | 2.31 | Yes |
| Vancomycin | Hematologic | 3232 | 4.52 (4.35-4.68) | 4.20 | 2.06 (2.01-2.11) | 4.06 | Yes |
| Vancomycin | Hepatotoxicity | 1389 | 3.58 (3.40-3.78) | 3.49 | 1.79 (1.72-1.87) | 3.32 | Yes |
| Vancomycin | Nephrotoxicity | 6675 | 11.86 (11.55-12.18) | 9.86 | 3.28 (3.24-3.31) | 9.51 | Yes |
| Vancomycin | Neurotoxicity | 686 | 1.21 (1.12-1.30) | 1.20 | 0.27 (0.16-0.37) | 1.13 | No |
| Vancomycin | Ototoxicity | 300 | 1.76 (1.57-1.97) | 1.75 | 0.81 (0.65-0.97) | 1.59 | No |
| Vancomycin | Skin/Allergy | 6697 | 5.59 (5.44-5.74) | 4.74 | 2.24 (2.20-2.27) | 4.62 | Yes |
| Vancomycin | Tendon/MSK | 115 | 1.39 (1.16-1.67) | 1.39 | 0.47 (0.21-0.74) | 1.19 | No |

Note for Table S16 — BH-FDR correction was applied to chi-square p-values. All 103 robust signals had q < 0.05. Robust signals were defined by all four measures simultaneously meeting their thresholds (ROR lower 95% CI > 1; PRR ≥ 2 with χ² ≥ 4; IC lower 95% CI > 0; EB05 ≥ 2; exposed-event count a ≥ 3), where the PRR χ² ≥ 4 cutoff is the Evans criterion, i.e., the conventional integer rounding of the exact chi-square critical value at the two-sided 5% significance level for one degree of freedom (χ² = 3.84 at α = 0.05, df = 1).

**Table S17. Label-Concordance and BJI-Contextual Interpretation of Prioritized Signals**

The table distinguishes label- or literature-concordant monitoring priorities from novel causal claims.

| **Drug** | **Signal** | **Label/literature concordance** | **BJI-contextual interpretation** |
| --- | --- | --- | --- |
| Vancomycin | Nephrotoxicity | Concordant with established renal monitoring guidance | Label-concordant renal monitoring priority, not a novel toxicity |
| Linezolid | Hematologic toxicity | Concordant with known myelosuppression/thrombocytopenia risk | Label-concordant CBC monitoring priority during prolonged therapy |
| Ceftaroline | Hematologic toxicity | Partly concordant with hematologic events and prolonged-exposure neutropenia reports | Prominence in prolonged BJI therapy is the incremental observation |
| Ertapenem | Neurotoxicity | Concordant with label-described seizures/CNS events, especially with renal impairment | Monitoring-priority hypothesis during extended therapy, not incidence/causality evidence |
| Daptomycin | Myotoxicity | Concordant with creatine-kinase elevation/rhabdomyolysis monitoring | PT-level analysis clarifies that myotoxicity was present despite tendon/MSK attenuation |
